# Phylogenetic, antigenic and biological characterization of pigeon paramyxovirus type 1 circulating in China

**DOI:** 10.1186/s12985-017-0857-7

**Published:** 2017-09-29

**Authors:** Xusheng Qiu, Chunchun Meng, Yuan Zhan, Shengqing Yu, Shichao Li, Tingting Ren, Weifeng Yuan, Shuqin Xu, Yingjie Sun, Lei Tan, Cuiping Song, Ying Liao, Zhuang Ding, Xiufan Liu, Chan Ding

**Affiliations:** 1 Shanghai Veterinary Research Institute, Chinese Academy of Agricultural Sciences, Shanghai, 200241 People’s Republic of China; 2 Key Laboratory of Animal Infectious Diseases, Yangzhou University, Yangzhou, Jiangsu China; 3 Jiangsu Co-innovation Center for Prevention and Control of Important Animal Infectious Diseases and Zoonoses, Yangzhou, Jiangsu China; 4 Laboratory of Infectious Diseases, College of Veterinary Medicine, Jilin University, Changchun, Jilin China

**Keywords:** Newcastle disease virus, Pigeon, Virulence, Phylogenetic analysis, Antigenic differences

## Abstract

**Background:**

For many years, ND has been one of the most important infectious pigeon diseases in China. In recent years, a high mortality has been observed in ND-infected pigeons in China. Mortality is from 40% to 80% or 100% in some cases.

**Methods:**

The full-length genomes of four pigeon paramyxovirus type 1 (PPMV-1) strains, which were isolated from infected pigeons in China in 2012 and 2013, were sequenced and analyzed to determine the phylogenetic characteristics of PPMV-1 circulating in pigeons of China in recent years. Furthermore, cross hemagglutination inhibition and cross virus neutralization assays, as well as animal experiments were conducted to determine the antigenicity and pathogenicity of those viruses. Proteolytic cleavage sites (residues 112–117) of the F proteins were identified as the typical virulence motif, ^112^RRQKR↓F^117^ for all four PPMV-1 strains investigated.

**Results:**

Phylogenetic analysis based on sequences of complete genomes and F gene revealed that the four PPMV-1 isolates and most of recent isolates in China were highly homologous to European isolates from 1998 to 2011. All those isolates were clustered in one clade of genotype VI NDV, termed as subgroup 4bii f. The *R* value was calculated based on cross hemagglutination inhibition and cross virus neutralization results, and confirmed antigenic difference of the PPMV-1 strains isolated in 2013 from the LaSota vaccine strain. Several mutations were identified in the surface glycoproteins F and HN, which probably gave rise to those antigenic differences.

**Conclusion:**

Our result suggested that the PPMV-1 strain prevailing in China in the last decade diverged from a common ancestor and was presumably transmitted from Europe. PPMV-1 isolates displayed obvious antigenic differences from vaccine strain LaSota. Even though PPMV-1 did not cause high mortality in experimental pigeons, the infected pigeons were exhibiting viral shedding for 3 weeks after infection, suggesting PPMV-1 is a potential threat to NDV control worldwide.

**Electronic supplementary material:**

The online version of this article (10.1186/s12985-017-0857-7) contains supplementary material, which is available to authorized users.

## Background

Newcastle disease (ND) in pigeons is responsible for one of the most severe recent infectious pigeon diseases in China [1–3]. The causative agent of ND in pigeons is Newcastle disease virus (NDV), also called pigeon paramyxovirus type 1 (PPMV-1). NDV is taxonomically classified into the genus *Avulavirus*, subfamily *Paramyxovirinae*, family *Paramyxoviridae*, order *Mononegaviriales* [4, 5]. The NDV genome consists of a single-stranded, negative-sense RNA, containing six genes in the sequence of 3′-NP-P-M-F-HN-L-5′. Six structural proteins are encoded as nucleoprotein (NP), phosphoprotein (P), matrix protein (M), fusion protein (F), hemagglutinin-neuraminidase (HN) and large protein (L) [6]. In addition, two nonstructural proteins, V and W, are generated from the P gene by RNA editing [7, 8].

NDV isolates are phylogenetically divided into two major classes, class I and class II [9, 10]. Class I NDV isolates are widely separated from healthy birds, and nearly all are nonvirulent [10–14]. NDVs belonging to class I are characterized by a 15,198-nt genomic RNA. By contrast, class II NDVs have two genome types. According to the classification system proposed by Claudio L. Afonso et al. in 2012, class I viruses contain a single genotype whereas class II NDV isolates can be effectively divided in 18 genotypes (I - XVIII) [15]. Genotype I-IV viruses are early lineage (before 1960) with a genome size of 15,186 nt. Genotype V-VIII are recent lineage (after 1960) with a genome of 15,192 nt [4, 10, 16–18]. Most pigeon-derived virulent NDV isolates are class II genotype VI, which was further divided into VIa - VIg. Subtype VIa strains, with the representative strain Iraq AG68, were isolated from Middle Eastern epizootics in the 1960s and subsequently spread to Africa and Asian countries [19]. Subtype VIb NDV of pigeon origin is widely accepted to be responsible for the third ND pandemic during the 1980s. In a Chinese outbreak around 2000, subgenotype VIb [20], VIf and VIg [19] were all identified from pigeons, chickens and other fowls, whereas in the last decade, little VIf and VIg outbreaks have been reported [21].

Based on the pathogenicity of ND strains in chickens, NDV isolates are generally classified as lentogenic (nonvirulent), mesogenic (intermediate virulent) and velogenic (highly virulent), which are further classified as viscerotropic velogenic and neurotropic velogenic [22]. The virulence of NDV strains is reported to be mainly determined by cleavage site sequence of the F protein [23, 24]. NDV F protein is generated as precursor F0; when cleaved into disulfide-linked F1 and F2 polypeptides, F protein changes to be fusogenic and is crucial in mediating fusion of the viral envelope with cellular membranes. The consensus sequence of F protein cleavage sites of velogenic and mesogenic strains is ^112^(R/K)RQ(R/K)RF^117^. Common lenogenic strains have a monobasic amino acid motif at the same site, ^112^(G/E)(K/R)Q(G/E)R↓L^117^. Different F protein cleavage sites are substrates for different types of cellular proteases [25, 26]. The F protein of lentogenic strains is cleaved only by trypsin-like enzymes, which are confined to the respiratory and intestinal tracts. The F protein of virulent viruses such as genotype VI and VII strains are cleaved by ubiquitous and diverse host protease(s). Based on these findings, virulent NDV is defined by the World Organization for Animal Health as a virus of avian paramyxovirus-1 (APMV-1) with at least three multiple basic amino acids (arginine or lysine) residues at the C-terminus of the F2 protein (between residues 113 and 116) and phenylalanine at residue 117, which is the N-terminus of the F1 protein [22, 27].

For many years, ND has been one of the most important infectious pigeon diseases in China, but low mortality of this disease is widely reported in most countries. After infection with pigeon-origin genotype VI ND, pigeons showed clinical signs similar to neurotropic ND-infected chickens, with a morbidity from 30% to 70% and a mortality no more than 10% [28, 29]. In recent years, a high mortality has been observed in ND-infected pigeons in China. Mortality is from 40% to 80% or 100% in some cases [1–3]. Whether pigeon-origin NDV isolates in China are more pathogenic for pigeons remains unclear. In this study, we analyzed genomic sequences of four PPMV-1 strains recently isolated in China, furthermore, the virulence, antigenicity and potential threat of the viruses were evaluated.

## Methods

### Viruses and pathogenicity tests

Pigeon-origin NDV strains pi/SD/CH/0132/2012 (designated ND132 in this study), pi/SH/CH/0163/2012 (ND163), pi/SH/CH/0167/2013 (ND167), and pi/SH/CH/0168/2013 (ND168) were isolated from clinical specimens of diseased pigeons in Shanghai and Shandong Provinces in China in 2012 and 2013. F48E8 (eighth egg-passaged stock of F48), the standard ND challenge strain in China [19] and chicken vaccine strain LaSota were from the China Institute of Veterinary Drug Control (Beijing, China). All viruses were propagated in 9- to 11-day-old specific-pathogen-free (SPF) chicken embryonated eggs as described [30]. Fresh allantoic fluid was harvested from embryonated eggs dead between 24 and 120 h after inoculation, and kept at −80 °C. Mean death time (MDT) and intracerebral pathogenicity index (ICPI) tests were determined as previously described to determine the virulence of the four isolates [27].

### Viral RNA extraction, RT-PCR and full-length genome sequencing

The genomic RNA of ND132, ND163, ND167, and ND168 was extracted from the allantoic fluid of infected eggs using TRIzol reagent (Invitrogen, Carlsbad, CA, USA), according to the manufacturer’s instructions. RNA pellets were resuspended in 50 μL RNase-free water and reverse transcribed with 6-nt random primer with Mo-MLV reverse transcriptase (Promega, Madison, WI, USA). The cDNA was used as template to generate nine successive and overlapping DNA fragments by PCR using primer pairs specific for genotype VI NDV strains (Additional file 1: Table S1). Reverse transcription and PCR was performed without modification as described [31].

To determine the 3′- and 5′-ends of the viral genomes, rapid amplification of cDNA end (RACE) was performed as reported previously [32]. For 3′-RACE, genomic RNA was ligated with 5′-end-phosphorylated adaptor CL+ (Additional file 1: Table S1) using T4 RNA ligase (Fermentas, Shenzhen, China) in the presence of RNasin (Takara Biotechnology, Dalian, China) at 1 U/μl. After denaturation at 75 °C for 15 min, ligated RNA was transcribed with anti-adaptor CL- using Mo-MLV Reverse Transcriptase, which was complementary to adaptor primer CL+. The 3′-end of the genome was PCR-amplified with CL- and 3TSR, which was specific for the NP gene.

For 5′-RACE, first-strand cDNA was reverse-transcribed from viral RNA using specific primer 5TLF. After treatment with an equal volume of 0.6 N NaOH for 20 min at 60 °C to hydrolyze mRNA, cDNA was ligated with adaptor CL+ using T4 RNA ligase. Adaptor-ligated cDNA was PCR-amplified using primer 5TLF and anti-adaptor primer CL- and 1 μl PCR products was used for a second-round hemi-nested PCR reaction with primer CL- and specific primer 5TSF to generate the 5′ end of the viral genome.

PCR products were extracted from agarose gels and ligated into the TA cloning system (Promega). Ligation products were transferred into *Escherichia coli* DH5α and identified by restriction enzyme analysis. At least five clones for each segment were sent to Sangon Biotechnology (Shanghai, China) for sequencing.

### Sequence analysis

Prediction of amino acid sequences, alignment of sequences and phylogenetic analysis was performed using the MegAlign program in the Lasergene package (DNASTAR Inc. Madison, WI, USA). Phylogenetic trees were constructed using MEGA version 7 [33]. The genome sequences of 37 reference NDV strains, and the F gene sequences from 360 genotype VI NDV isolates and 24 other genotype representative strains were from EMBL/GenBank [31, 34]. All data and accession numbers of NDV strains used in this study are listed in Additional file 2: Table S2.

### Cross hemagglutination inhibition and cross virus-neutralization tests

Cross hemagglutination inhibition test was performed as described [3] using four hemagglutinating units of LaSota, ND167 or ND132 virus and antisera against the NDV strains. For cross virus-neutralization (VN) tests, the 50% embryo infectious dose (EID_50_) value of LaSota, ND167 and ND132 viruses was determined in advance by inoculation at a 10-fold dilution into 10-day-old SPF embryonated chicken eggs. Antisera against LaSota, ND167 and ND132 were serially double-diluted and mixed respectively with each NDV strain at 100 EID_50_/100 μl. After incubation at 4 °C for 1 h, mixtures were injected into five, 10-day-old SPF embryonated chicken eggs to determine neutralization levels. Results of cross-VN and cross-HI tests were expressed as average and standard deviation (SD) and *R*-value was calculated using the formula of Archetti and Horsfall [35].

Antisera used in this study were obtained from chickens and pigeons according to a previous report [1], with minor modification. First, 30-day-old SPF chickens and 50-day-old clinically healthy pigeons were intramuscularly injected with inactive oil-emulsified LaSota, ND167 or ND132 strains (10^6^ EID_50_ per chick and 10^5^ EID_50_ per pigeon). After 2 weeks, booster doses were administered. Birds were exsanguinated for serum collection 1 week after last inoculation. All sera were inactivated at 56 °C for 30 min and stored at −80 °C.

### Animal experiment design

A total of 56 clinically healthy 40-day-old pigeons without any antibodies against ND were used and housed in negative pressure isolators (Fengshi Group, Suzhou, China). Experiments were carried out in strict accordance with the recommendations in the Guide for the Care and Use of Laboratory Animals of Shanghai Veterinary Research Institute, Chinese Academy of Agricultural Sciences. Samples were collected from three pigeons randomly chosen before experiments and all pigeons after experiments were detected for other pigeon viruses by multiplex PCR [36]. All pigeons were free of pigeon herpesvirus, adenovirus and circovirus (data not shown).

Pigeons were randomly divided into eight groups of seven birds per group: groups A1, A2, A3, B1, B2, B3, PC and NC. All pigeons were inoculated with 0.2 ml 10^6^ EID_50_ virus via intravenous injection (groups A1 and B1), intramuscular injection (groups A2 and B2) or oral administration (groups A3 and B3). The titers of undiluted virus stock of ND132, ND167 and F48E8 were determined as 10^7.3^ ELD_50_/0.2 ml, 10^7.9^ ELD_50_/0.2 ml and 10^8.3^ ELD_50_/0.2 ml respectively. Pigeons in a positive control (PC) group were intramuscularly injected with 10^6^ EID_50_ F48E8; pigeons in negative control (NC) group were inoculated with sterile PBS (pH 7.2). Immediately after virus inoculation, all pigeons were kept in negative pressure isolators (Fengshi Group), under controlled laboratory and biosafety conditions. Pigeons were observed twice a day for 42 days, and any abnormal posture or behavior was recorded. Oral and cloacal swabs were collected at 5, 7, 10, 14, 21, 28, 35 and 42 days post infection (dpi) from experimental pigeons. Collected swabs were placed in 1.0 ml sterile PBS pH 7.2 and kept in −80 °C. Virus shedding was determined by virus isolation from swabs, using three SPF chicken eggs per sample.

## Results

### Sequence characteristics of the four PPMV-1 isolate genomes

**Fig. 1:**
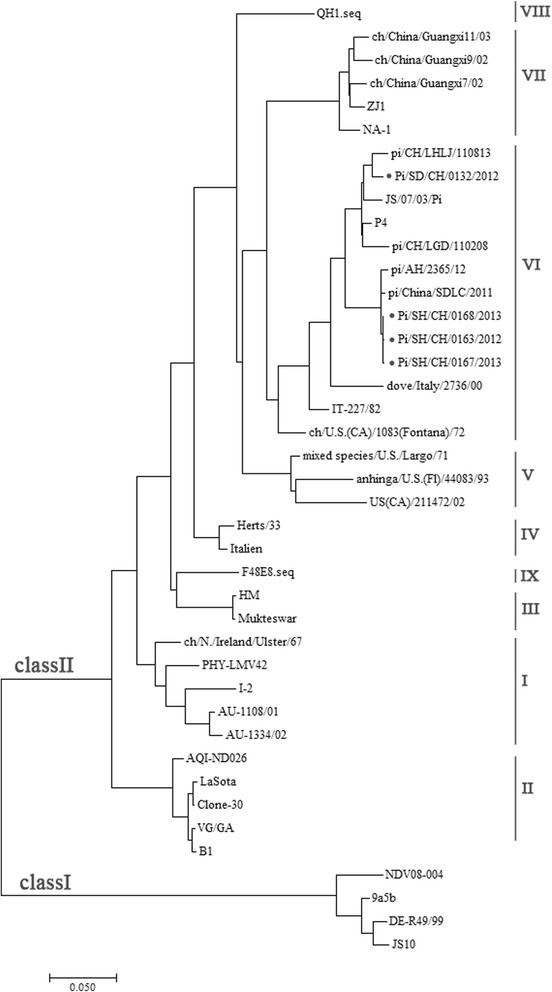
Phylogenetic tree based on full-length nucleotide sequences of NDV strains. The tree of 41 nucleotide sequences was constructed using MEGA7 [33]. Strain genotypes are marked on the right. The GenBank accession numbers of the reference NDV strains are listed in Additional file 2: Table S2. *, the strains investigated in this study

RT-PCR was performed for amplification of the expected nine overlapping fragments and the 5′ and 3′ ends of the genomes using respective primers in Additional file 1: Table S1. Amplified products were sequenced, aligned and assembled to obtain the full-length genome of four PPMV-1 strains. Sequences were submitted to EMBL/GenBank with the following accession numbers: KT163261 for ND163, KT163262 for ND167, KT163263 for ND168 and KT163264 for ND132. All the four genomes were 15,192 nucleotides (nt) in length, as reported for all other genotype VI NDV strains [27]. Compared with the genome of genotype I, II, and III NDV strains, all four isolates had a 6-nt insertion, CCCCAA, at position 1647 of the viral genome in the 5′-noncoding region of the NP gene. Phylogenetic analysis was performed using the complete genome sequences of the four NDV isolates, all of which were characterized as genotype VI (Fig. 1).

The proteolytic cleavage site motif (residues 112–117) of the F protein from the four PPMV-1 isolates was determined as ^112^RRQKR↓F^117^, the typical virulence motif [27]. Six predicted *N*-glycosylation sites, ^85^NRT^87^, ^191^NNT^193^, ^366^NTS^368^, ^447^NIS^449^, ^471^NNS^473^ and ^497^NTS^499^, were found in the F protein of all four isolates. The HN of the four PPMV-1 isolates was sequenced as 571 amino acid (aa) with five predicted *N*-glycosylation sites at ^119^NNS^121^, ^341^NNT^343^, ^433^NKT^435^, ^481^NHT^483^ and ^508^NIS^510^; which are conserved in PPMV-1 strains [37].

The nucleotide and amino acid sequences of the four PPMV-1 isolates were compared. In general, the six coding-region sequences showed very low divergence. The six genes of three Shanghai NDV isolates, ND163, ND167, and ND168, shared more than 99.9% identity for nucleotides and amino acids. ND132 showed lower homology to the three Shanghai NDV isolates. Shared nucleotide identities for ND132 and ND167 were 96% for the NP gene, 92.9% for P gene, 94.2% for M gene, 95.7% for F gene, 94.4% HN gene and 96% for L gene, and amino acid identities for ND132 and ND167 were 98.4% for NP protein, 93.2% for P protein, 93.2% for M protein, 97.3% for F protein, 96.9% for HN protein, and 98.7% for L protein. According to Basic Local Alignment Search Tool (BLAST) of NCBI, both full-length genomes of ND132 and ND167 showed a very high similarity to PPMV-1 strains isolated in China in recent decades. The Chinese strains with highest homology were pi/CH/LGD/110947 (accession number JX486556.1, 99.0% compared with ND132) and pigeon/China/SDLC/2011 (accession number JQ979176.1, 99.5% compared with ND167), both of which were isolated in China in 2011 [38]. The foreign strains that shared highest nucleotide homology were PPMV-1/Belgium/11–08304/2011 (JX901123.1) which had 99.0% identity with the three Shanghai NDV isolates.

### Phylogenetic analysis of pigeon-origin NDV isolates prevailing in China

**Fig. 2:**
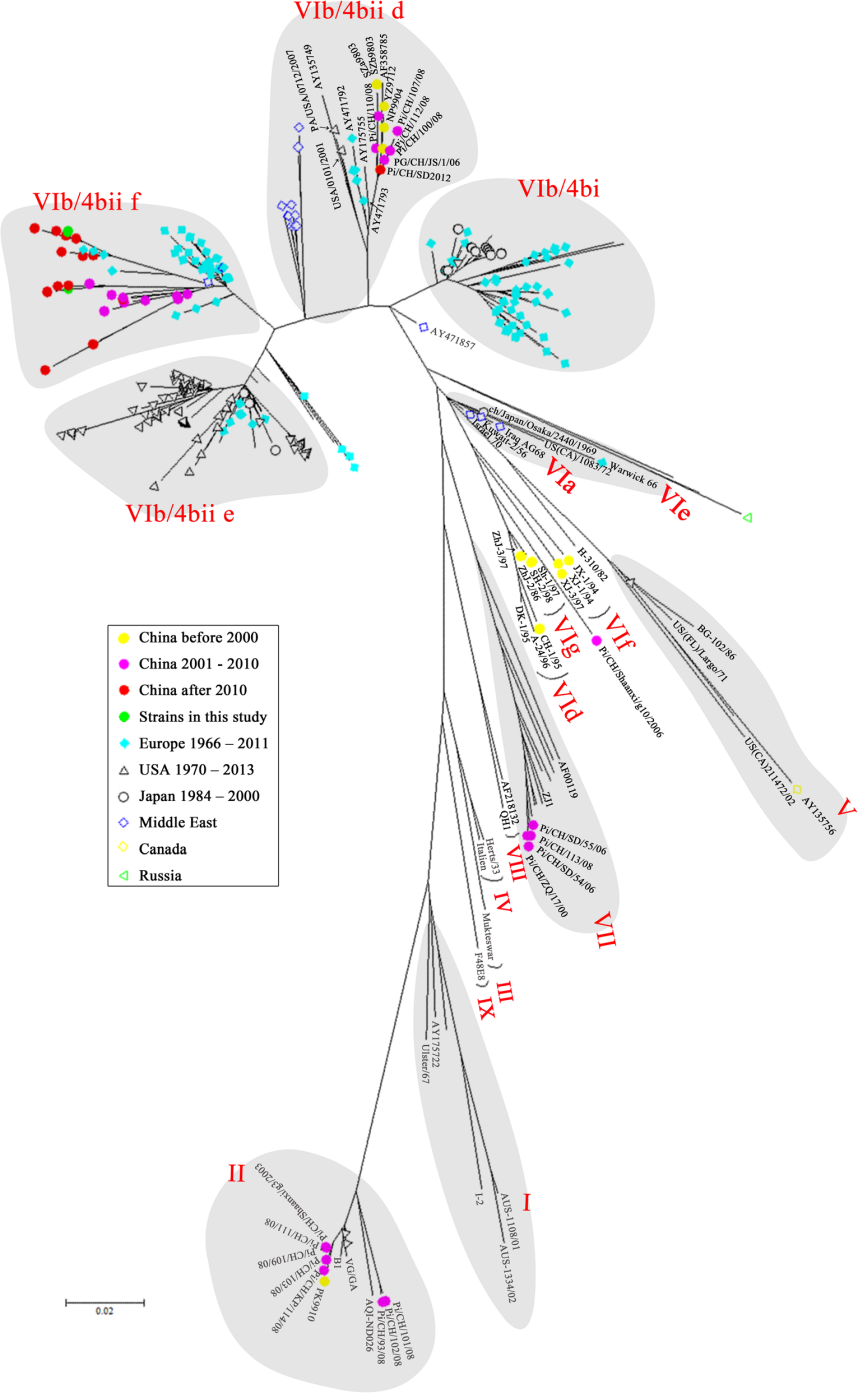
Unrooted maximum likelihood radial phylogram of the isolate genotypes. The tree was constructed on a 374-nt section of the F gene sequences from 360 genotype VI NDV isolates and 24 other genotypes representative strains in GenBank, and relevant evolutionary analyses were conducted in MEGA7 [33]. The tree is to scale, with branch lengths measured in number of substitutions per site. Names of each PPMV-1 strain are not shown but represented by colored symbols. Genotypes and groups of each clade are labeled. All data and accession numbers for reference F gene sequences of the isolates and strains were from EMBL/GenBank based on established phylogenic analysis (Additional file 2: Table S2)

Further phylogenetic analysis based on the variable region sequences (nt 47–420) of the F gene from 384 APMV-1 isolates showed that the four PPMV-1 isolates in this study were categorized as genotype VIb (Fig. 2). For this phylogenetic tree, nearly all pigeon-origin strains available in GenBank were collected. The tree displayed a relationship of the genotypes of NDV isolates and the time they were isolated. While subgenotype VIb [20], VIf and VIg [19] pigeon NDV were commonly isolated before 2000, VIb subtype NDV were predominantly identified from Chinese pigeons in China in recent two decades. Only one strain, pi/China/Shaanxi/g10/2006 [21], shared high nucleotide homology with subgenotype VIf and VIg.

In 2004, Aldous et al. systematically analyzed the phylogenesis of genotype VI NDV strains and divided the VIb subgenotype into groups 4bi and 4bii; the latter was further classified into clades d, e, f [34]. PPMV-1 isolates in China did not belong to group 4bi and clade 4bii e, and had further genetic distance from VIb NDV isolates in North America and Japan (Fig. 2).

**Fig. 3:**
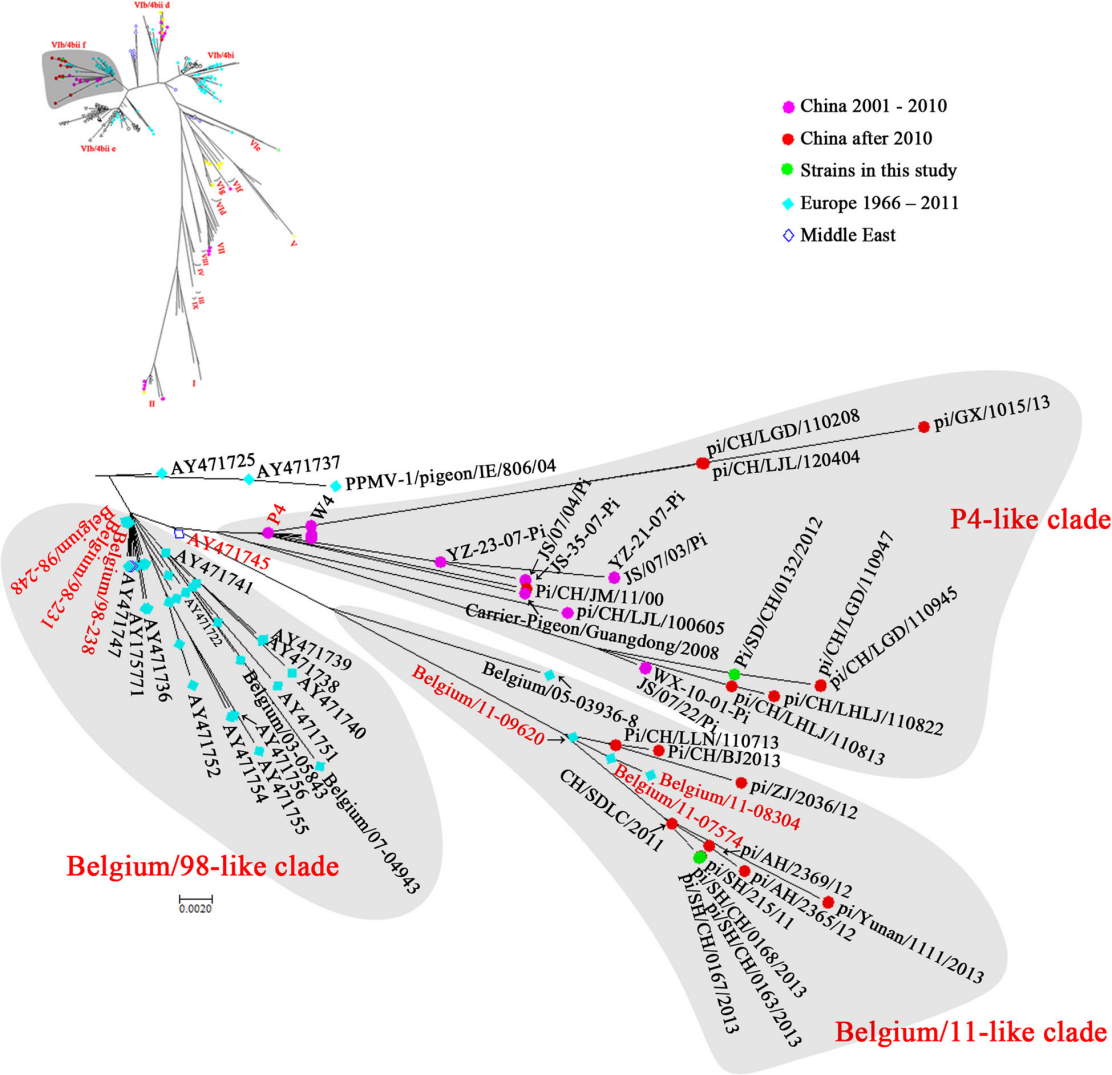
Unrooted maximum likelihood radial phylogram of the subgroup VIb/4bii f. The tree was constructed on a 374-nt section of the F gene sequences from 176 PPMV-1 isolates. Groupings of each clade are labeled

Most of the PPMV-1 isolates in China after 2000 (Fig. 2) were classified into clade 4bii f, which was further divided into three clades (Fig. 3). Our NDV isolate ND132 and other NDV isolates in distant provinces such as JS/07/03/Pi, pi/GX/1015/13 and pi/CH/LGD/110945, were highly homologous and in the same clade as P4 and W4; these were isolated from wild water birds in South China and deduced to be from Europe around 1999 [39]. The other Chinese isolates shared higher homology with Belgium/11–09620, Belgium/11–08304 and Belgium/11–07574, and showed further genetic distance from P4 and W4. This result suggested they may be directly derived from Europe but not from the local Chinese epidemic strains.

To further determine the homology of the four NDV isolates with previous isolates, the nucleotide and amino acid homologies of six viral genes were compared. Similar to the phylogenetic analysis, ND132 showed highest homology with Belgium/11–09620 (Additional file 3: Table S3). The other three Shanghai isolates, ND163,ND167 and ND168 were closest to P4 strain (Additional file 4: Table S4). No virulence-related differences were found between the European strains and our four isolates, which can be assumed to be representative of PPMV-1 prevailing in China in recent decade.

Based on sequence alignment, amino acid differences in surface glycoproteins between recent PPMV-1 isolates and the classic vaccine strain LaSota were identified. Specific substitutions for ND167 and ND132 are in Table 1. In the F protein of Belgium/11-like PPMV-1 strains, five specific substitutions were found: M14 T, P28S, V179I, A203S and V506I; only one specific substitution, I432V, was found in P4-like PPMV-1 strains. When the HN protein sequences were aligned and compared, I59V, A66V, L74I, T232 N, S432H, A497T and D569E were found in Belgium/11-like PPMV-1 strains; I84V and A145V were in P4-like PPMV-1 isolates. For all substitutions, V179I and V506I of F protein were located in the heptad repeat region a (HRa, aa 143–185) and transmembrane domain (aa 501–521); N263 K and D569E substitutions in Belgium/11-like PPMV-1 strains and N263R in P4-like PPMV-1 isolates were identified in neutralizing epitopes [3].

**Table 1:** Specific amino acid substitutions in F and HN proteins of endemic PPMV-1 strains in China compared to LaSota. Amino acids that are the same as LaSota are indicated by “-”

Subgenotype	clade	strains	F protein												HN protein											
			6	8	13	14	16	28	179	203	259	432	506		59	66	74	84	121	145	232	263	309	432	497	569
II		LaSota	S	K	M	M	T	P	V	A	Q	I	V		I	A	L	I	I	A	T	N	D	S	A	D
VIb/4bii f	Belgium/11-like	pi/SH/CH/0163/2012	H	R	P	T	I	S	I	S	H	–	I		V	V	I	–	–	I	N	K	–	H	T	E
		pi/SH/CH/0167/2013	H	R	P	T	I	S	I	S	H	–	I		V	V	I	–	–	I	N	K	–	H	T	E
		pi/SH/CH/0168/2013	H	R	P	T	I	S	I	S	H	–	I		V	V	I	–	–	I	N	K	–	H	T	E
		pi/YN/1111/13	H	R	P	T	I	S	I	S	H	–	I		V	V	I	–	–	I	N	K	–	H	T	E
		pi/AH/2365/12	H	R	P	T	I	S	I	S	H	–	I		V	V	I	–	–	I	N	K	–	H	T	E
		Belgium/11–09620/2011	H	R	L	T	I	S	I	S	H	–	I		V	V	I	–	–	I	N	K	–	H	T	E
		Belgium/11–07574/2011	H	R	L	T	I	S	I	S	H	–	I		V	V	I	–	–	I	N	K	–	H	–	E
	P4-like	P4	H	R	L	–	I	L	–	T	H	V	–		–	–	–	V	V	V	–	K	N	Y	–	–
		pi/SD/CH/0132/2012	H	G	L	–	V	L	–	T	–	V	–		–	–	–	V	V	V	–	R	N	Y	–	–
		JS/07/22/Pi	H	R	L	–	V	L	–	T	–	V	–		–	–	–	V	V	V	–	R	N	Y	–	–
		pi/CH/LHLJ/110822	H	R	L	–	V	L	–	T	–	V	–		–	–	–	V	V	V	–	R	N	Y	–	–
		pi/CH/LGD/110947	H	G	P	–	V	L	–	T	–	V	–		–	–	–	V	V	V	–	R	N	Y	–	–
	Belgium/98-like	Belgium/98–238/1998	Y	R	L	–	I	L	–	T	–	V	–		–	–	–	–	V	I	–	K	N	Y	–	–
		Belgium/98–248/1998	Y	R	L	–	I	L	–	T	–	V	–		–	–	–	–	V	I	–	K	N	Y	–	–
		Belgium/07–04943/2007	Y	R	L	–	I	L	–	T	–	V	–		–	–	–	–	V	I	–	K	N	Y	–	–
VIb/4bii d		Pigeon/China/SD2012	–	R	P	–	I	–	–	T	H	–	–		–	T	–	–	V	T	–	K	–	Y	–	–
VIb/4bi		IT-227/82	–	R	L	T	I	–	–	T	H	–	–		–	T	–	–	V	T	–	K	–	H	–	G
VIe		US(CA)/1083 Fontana/72	–	R	L	–	I	L	–	T	–	–	–		–	T	–	–	V	T	–	K	–	Y	–	–

### Cross hemagglutination inhibition and cross virus neutralization assays

**Fig. 4:**
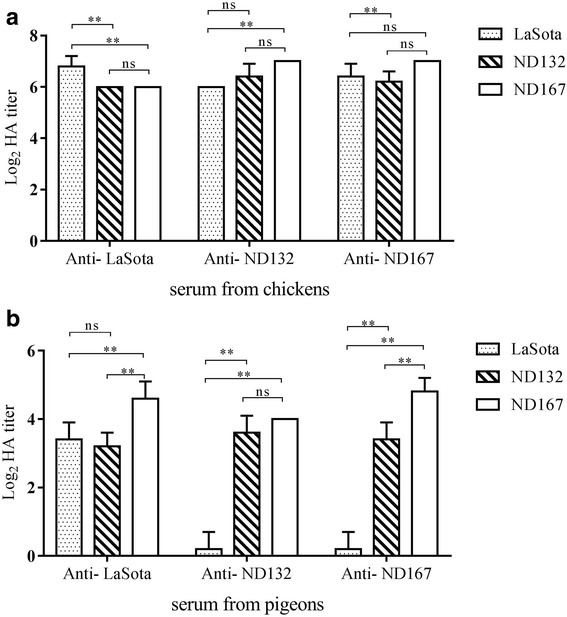
Cross hemagglutination inhibition values for anti-NDV chicken sera (**a**) and pigeon sera (**b**) against Lasota, ND167 and ND132. Data are presented as mean ± standard deviation. The statistical differences between each group of values were determined by two-way ANOVA and labeled in the figures. (* *P* < 0.05; ** *P* < 0.01; ns, no significant difference)

To determine the reactivity of anti-NDV serum with different isolates, cross-HI assay was carried out. Antiserum from chickens reacted with both heterologous and homologous antigens and all HI titers using homologous antigens were slightly higher than titers using other antigens (Fig. 4). *R* values for comparisons to the LaSota strain were 0.669 for ND132 and 0.616 for ND167, suggesting minor antigenic differences of the epidemic PPMV-1 isolates with the commonly used LaSota vaccine. Reactivity differences for pigeon sera with different isolates were more obvious. Anti-ND132 and anti ND167 pigeon serum rarely reacted with LaSota since HI titers of those sera with homologous antigens were 2^3.6^ and 2^4.8^. *R* values for LaSota strain were 0.287 for ND132 and 0.308 for ND167 using pigeon-derived serum, indicating definite antigenicity differences between these strains.

Chicken antisera against LaSota, ND132 and ND167 were used in cross-VN assays to further determine antigenicity differences among ND132, ND167 and LaSota (Table 2). The 50% endpoints were calculated according to the Reed and Muench method [40]. Against homologous strains ND132 and LaSota, neutralization titers of anti-ND167 serum were 1:189, 1:51 and 1:25. *R* values for comparisons to ND167 were 0.208 for LaSota and 0.412 for ND132, indicating striking antigenic differences of ND167 with other strains.

**Table 2:** Cross virus-neutralization titers for anti-NDV sera against LaSota, ND167 and ND132 in embryonated chicken eggs

Anti-serum	NDV strain		
	La Sota	ND132	ND167
Anti-LaSota	1:288	1:102	1:94
Anti-ND132	1:150	1:256	1:161
Anti-ND167	1:25	1:51	1:189

### Pathogenicity studies

ND132, ND163, ND167 and ND168 were subjected to pathogenicity index tests using chickens. The MDT of all four isolates was in 60–90 h, and the ICPI index was between 0.7 and 1.5 (Table 3) [41].

**Table 3:** Pathogenicity indexes for PPMV-1 isolates in this study

Strain	HA titer	MDT	ICPI
ND132	2^6^	66 h	0.95
ND163	2^7^	62 h	1.15
ND167	2^7^	60 h	1.21
ND168	2^7^	65 h	1.18

To determine the mortality of PPMV-1 prevailing in China in recent decade, pigeons were infected with ND132 in the Belgium-11-like clade and ND167 in the P4-like clade. At 5 dpi, infected pigeons were depressed and displayed ruffled feathers and transient fever. Pigeons infected with ND132 or ND167 recovered at 7 dpi, and showed no clinical symptoms. In F48E8 infected group, three pigeons died at 5 or 8 dpi. No apparent clinical signs were observed in pigeons of the negative-control group.

To determine viral shedding, oropharyngeal and cloacal swabs were taken on 5, 7, 10, 14, 21, 28, 35 and 42 dpi. The samples were subjected for NDV isolation using SPF embryonated eggs (Table 4). Virus shedding from pigeon throats was detectable from all seven infection groups at 5 dpi, especially in the two groups infected via intravenous injection. The highest shedding rates were observed from the throat at 7 dpi and cloaca at 10 dpi. Viral shedding reduced at 14 dpi and ceased at 21 dpi. Mock-inoculated pigeons remained virus negative throughout the experiments.

**Table 4:** Viral shedding of PPMV-1 from the oral cavity and cloaca of experimentally infected pigeons.﻿ Pigeons in groups A1, A2 and A3 were infected with 10^6^ EID_50_pi/China/SD/2012/132; pigeons in groups B1, B2 and B3 were infected with 10^6^ EID_50_ pi/China/SH/2013/167. Positive-control group (PC) birds were intramuscularly injected with 10^6^ EID_50_ F48E8; and negative-control group (NC) birds were inoculated with sterile PBS (pH 7.2)﻿.﻿ IV, intravenous injection; IM, intramuscular injection; IN, intranasal infection; IO, intraocular infection

Group		A1	A2	A3	B1	B2	B3	PC	NC
dpi		IV	IM	IN&IO	IV	IM	IN&IO	IM	IM
5	Oropharyngeal	0/7	0/7	2/7	2/7	1/7	2/7	2/6	0/7
	Cloacal	0/7	0/7	0/7	1/7	0/7	0/7	0/6	0/7
7	Oropharyngeal	3/7	1/7	2/7	3/7	6/7	1/7	6/6	0/7
	Cloacal	0/7	0/7	0/7	1/7	0/7	1/7	2/6	0/7
10	Oropharyngeal	3/7	4/7	0/7	1/7	3/7	1/7	3/4	0/7
	Cloacal	7/7	6/7	1/7	6/7	7/7	1/7	1/4	0/7
14	Oropharyngeal	0/7	0/7	0/7	1/7	0/7	0/7	1/4	0/7
	Cloacal	1/7	1/7	0/7	2/7	1/7	1/7	1/4	0/7
21	Oropharyngeal	0/7	0/7	1/7	0/7	0/7	0/7	0/4	0/7
	Cloacal	1/7	0/7	0/7	0/7	0/7	0/7	0/4	0/7
28	Oropharyngeal	0/7	0/7	0/7	0/7	0/7	0/7	0/4	0/7
	Cloacal	0/7	0/7	0/7	0/7	0/7	0/7	0/4	0/7
35	Oropharyngeal	0/7	0/7	0/7	0/7	0/7	0/7	0/4	0/7
	Cloacal	0/7	0/7	0/7	0/7	0/7	0/7	0/4	0/7
42	Oropharyngeal	0/7	0/7	0/7	0/7	0/7	0/7	0/4	0/7
	Cloacal	0/7	0/7	0/7	0/7	0/7	0/7	0/4	0/7

## Discussion

Four virulent PPMV-1 strains, ND132, ND163, ND167 and ND168, were isolated in Chinese pigeon farms; 70–80% of raised pigeons died from the outbreaks [41]. The genomes of the viruses were sequenced and subjected to phylogenetic analysis with sequences from 380 NDV strains isolated from China, Japan, the Middle East, Europe and North America. Based on analysis of six viral proteins, all four isolates were classified into subgenotype VIb and had high homology to most PPMV-1 strains isolated after 2000 from distinct provinces in China (Fig. 2, Additional file 3: Table S3 and Additional file 4: Table S4). In a Chinese outbreak around 2000, subgenotype VIb, VIf and VIg [19] were all identified from pigeons, chickens and other fowls, whereas in the last decade, little VIf and VIg outbreaks have been reported [21]. Subgenotype VIb NDV is the most common in pigeons worldwide in the past two decades, and isolation of other subgenotypes, such as VIa, VId, VIf and VIg is rarely reported. Our four PPMV-1 isolates, ND132, ND163, ND167 and ND168, had high homology to each other but not to Chinese VIb isolates (representative strain YZ9712) from 10 years ago.

In this study, we used the Aldous method to further classify genotype VI genotype strains, which was based on 374 nucleotides in F protein of PPMV-1 isolated from Asia, Europe and America from 1978 to 2002 [34]. Based on this typing method, subgenotype VIb was divided into six clades: a, b, c, d, e and f. The first three were termed 4bi and the others 4bii. Two groups of VIb PPMV-1 were frequently isolated in China from 1998 until 2013. These groups are 4bii f clade and 4bii d clade. Isolates in clade d span three continents from 1983 to 2001. Isolates in this clade have been inferred to originate from two isolates in 1995, one from a budgerigar in Turkey, the other from a pigeon in Germany [34]. In this clade, Chinese, European and American isolates clustered on a separate branch from Middle Eastern isolates. Chinese PPMV-1 in this clade were isolated one or two decades ago, with the exception of pi/CH/SD2012, which was isolated in the Shandong province of China in 2012 [37]. These results suggested that viruses in the 4bii d clade were not widespread in China in recent decade.

Our four PPMV-1 isolates and most strains isolated from China in recent decade were highly homologous to Belgian isolates from 1998 to 2011, all of which clustered in 4bii f. This clade contained European and Middle Eastern viruses isolated from pigeons between 1998 and 2011. Based on the phylogenetic analysis and isolation date, 4bii d was the predominant clade in China 20 years ago. The clade 4bii f viruses began to emerge in the late 2000s, when the clade 4bii d viruses began to decline.

PPMV-1 isolates in 4bii f mainly diverged into three branches, termed Belgium/98-like, P4-like and Belgium/11-like in this study. All three branches originated from PPMV-1 strains such as PPMV-1/Belgium/98–231/1998, PPMV-1/Belgium/98–238/1998 and PPMV-1/Belgium/98–248/1998 isolated in Belgium around 1998. ND132, ND163, ND167 and ND168 were classified into different branches of 4bii f. ND132 clustered with P4 and W4 on a separate branch of the 4bii f clade. Both P4 and W4 were isolated from wild birds in Guangdong province, south of China; P4 was from a wild pigeon in 2003 and W4 from a white-breasted water hen in 2005. Molecular dating based on different genes suggests that these viruses diverged from a common ancestor around 1999 [39]. Viruses belonging to this branch were continually isolated in distinct provinces of China from 2003 until 2013, suggesting P4-like PPMV-1 has long been circulating throughout China.

The genomic sequences of three Shanghai NDV isolates, ND163, ND167 and ND168, showed the highest homology to PPMV-1/Belgium/11–08304/2011 (99.02%) [42]. The nucleotide and amino acid identities of six viral proteins were even higher, at 99.04–100%. All other isolates in this branch were isolated in China since 2011. This result suggested that the viruses diverged from a common ancestor around 2011 and presumably were transmitted from Europe. In summary, our four isolates represented two groups of PPMV-1 prevailing in China in recent years. The P4-like group emerged around 1998 and the Belgium/11-like group around 2011.

Our cross-HI and cross-VN results showed antigenicity differences between the commonly used vaccine LaSota and current PPMV-1 strains in China, especially ND167, which is in a clade of the Belgium/11-like group. The *R* values of NDV167 compared with LaSota were 0.287 in cross-HI assays and 0.208 in cross-VN assays, suggesting that PPMV-1 strains prevailing in China were antigenically changing. The specific amino acid substitutions in the F and HN proteins of NDV167 may have led to major antigenicity differences, such as M14 T, P28S, V179I, A203S, V506I in F protein and I59V, A66V, L74I, T232 N, S432H, A497T, D569E in HN protein. Recent Chinese and European isolates of PPMV-1 diverged from a common origin and kept changing, probably due to immune pressure of the live NDV vaccines such as LaSota. These mutations led to antigenic variation and will probably result in the inefficiency of the vaccine in the foreseeable future.

Phylogenetic analysis of the entire genome of four PPMV-1 isolates in this study showed that the viruses prevailing in China in recent years originated from Europe, and had high identity with Belgian isolates from 1998 to 2011. No virulence-related mutations were found in the six viral genes of the current Chinese isolates compared to the Belgian isolates. To determine the virulence of those viruses, we measured pathogenicity to pigeons of ND132 in the P4-like group and ND167 in the Belgium/11-like group.

All pigeons infected with ND132 and ND167 showed transient clinical signs but no mortality when pigeons were infected with PPMV-1 via oral administration, intramuscular injection, or intravenous injection. In contrast, the chicken-origin virulent NDV strain F48E8 caused 100% morbidity; three pigeons died and four survived. The genotype VIb PPMV-1 appeared to be less pathogenic to pigeons than the chicken-origin strain F48E8 did, which was consistent with the ICPI values. In previous studies, the pathogenicity of VIb to chickens was determined to be mesogenic [37, 38]. Thus, the 4bii PPMV-1 strains used in this study displayed the same pathogenicity as reported for European isolates [43–46]; pigeons seemed to be resistant to those viruses. In other words, no definitive evidence confirmed a marked increase in the pathogenicity of recent Chinese isolates to pigeons. The high mortality clinically observed in China may not be caused simply by genetic variation of endemic strains; the influence of the pigeon lineage, co-infection with other pathogenic microorganisms, feeding environment, and even local climate need to be reconsidered.

A notable problem is viral shedding from infected pigeons. Our results showed that infected pigeons continued to shed, even at 3 weeks after infection, suggesting healthy pigeons could be PPMV-1 carriers. Based on epidemiological surveys, genotype VIb strains were identified from healthy-looking pigeons and doves. All isolates had a virulence motif of ^112^RRQKRF^117^ in the F protein [3, 47]. The virulent isolates from symptomless pigeons from 2011 to 2013 in live poultry markets clustered in the 4bii f clade [3] and were highly homologous to our isolates. We propose that recent 4bii velogenic PPMV-1 isolates tended to develop a symbiotic relationship with pigeons so that infected pigeons showed no symptoms, became healthy carriers and shed occasionally. Previous studies showed that the same PPMV-1 strains were equally pathogenic to chickens and pigeons [48, 49]. The disease in pigeons occurs because of spread from diseased chicken flocks and from domesticated or feral pigeons to poultry [50]. Pigeon-origin subtype VIb viruses are widely considered to be responsible for the third pandemic of the 1980s. As reported in this study, current PPMV-1 isolates are gradually changing and giving rise to antigenic differences from vaccine strain LaSota, suggesting that PPMV-1 will be an imminent threat to NDV control worldwide.

## Conclusion

In this study, the full-length genomes of four virulent PPMV-1 strains, ND132, ND163, ND167 and ND168, were sequenced and subjected to phylogenetic analysis. Phylogenetic analysis revealed that the four PPMV-1 isolates were highly homologous to Belgian isolates from 1998 to 2011, as most of the recent isolates in China did, which suggested that the PPMV-1 strains prevailing in China in the last decade diverged from a common ancestor and was presumably transmitted from Europe. Several mutations were identified in the surface glycoproteins F and HN, which probably gave rise to antigenicity differences of PPMV-1 strains isolated in 2013 from LaSota vaccine strain. In this study, PPMV-1 did not cause high mortality in experimental pigeons, however, infected pigeons were exhibiting for viral shedding for3 weeks after infection, suggesting PPMV-1 is a potential threat to NDV control worldwide.

## Additional files

Additional file 1: **Table S1.** Primers used in this study to generate overlapping PCR fragments from the genome of pigeon-origin NDV isolates. (DOCX 16 kb)

Additional file 2: **Table S2.** Reference NDV strains in this study. (DOCX 52 kb)

Additional file 3: **Table S3.** Gene homology of NDV132 to other PPMV-1 strains (DOCX 18 kb)

Additional file 4: **Table S4.** Gene homology of NDV167 to other genotype VI NDV strains. (DOCX 17 kb)

## Abbreviations

aa: Amino acid
APMV-1: Avian paramyxovirus-1
CAAS: Chinese Academy of Agricultural Science
dpi: Day post infection
EID_50_: 50% embryo infectious dose
F: Fusion protein
HI: Hemagglutination inhibition
HN: Hemagglutinin-neuraminidase
ICPI: Intracerebral pathogenicity index
L: Large protein
M: Matrix protein
MDT: Mean death time
ND: Newcastle disease
NDV: Newcastle disease virus
NP: Nucleoprotein
nt: Nucleotides
P: Phosphoprotein
PBS: Phosphate-buffered saline
PCR: Polymerase chain reaction
PPMV-1: Pigeon paramyxovirus type 1
SD: Standard deviation
VN: Virus neutralization
