# Supplementary material for: Phylogenetic, antigenic and biological characterization of pigeon paramyxovirus type 1 circulating in China
Source: Virol J. 2017 Sep 29;14:186. doi: 10.1186/s12985-017-0857-7 (PMC5622419; doi:10.1186/s12985-017-0857-7)
Supplement: Supplementary file 1 — Table S1. Primers used in this study to generate overlapping PCR fragments from the genome of pigeon-origin NDV isolates. (DOCX 16 kb) [file 12985_2017_857_MOESM1_ESM.docx]

# Table S1 Primers used in this study to generate overlapping PCR fragments from the genome of pigeon-origin NDV isolates

| **Fragment designation** | **Primer sequence (5'-3')** | **Position**  **(nt)** | **Expected**  **size (bp)** |
| --- | --- | --- | --- |
| VIF1 | 5'- ACC AAA CAG AGA ATC KGT GAG TTA CGG -3' | 1 - 28 | 2120 |
| VIR2120 | 5'- TAG CTG TTT GTC CGG TCT GTC CTG TTG GTC -3' | 2120 - 2091 |  |
| VIF1892 | 5'- AAT GGC CAC CTT TAC CGA TGC T -3' | 1892 - 1913 | 1898 |
| VIR3789 | 5'-CCA GGG ATC TTC TCG GGT GCT TTC A-3' | 3789 - 3765 |  |
| VIF3531 | 5'-ACG ACA ATC CCA GAC ACG AGT TA-3' | 3531 - 3553 | 1765 |
| VIR5285 | 5'-AAG TAA TCC ATG TTG CCA CCA G-3' | 5295 - 5274 |  |
| VIF5126 | 5'- ACG GCA CGA GAA TTG GAC TGT A -3' | 5126 - 5147 | 1916 |
| VIR7041 | 5'- AAG TGC TAA GTA TTG ATG TGA GTG TG -3' | 7041 - 7016 |  |
| VIF6818 | 5'- GGG GGA TAG GTA AAG AAC TCA TAG -3' | 6818 - 6841 | 1974 |
| VIR8791 | 5'- AAC ATG CGT GCA CAG CTT GGC GAA CAG -3' | 8791 - 8765 |  |
| VIF8554 | 5'- GAA AAT ACT TGA ATC TGC CAC TC -3' | 8554 - 8576 | 1735 |
| VIR10288 | 5'- AAC TCT TCG ACG ATT CTT GCT CT -3' | 10288 - 10266 |  |
| VIF10055 | 5'- TTG ACA AAG AAA TTA AGG AAC TGC -3' | 10055 - 10078 | 1840 |
| VIR11894 | 5'- ACC AAG TAA ACT GTT CAT CTC CGC TGT C -3' | 11894 - 11867 |  |
| VIF11506 | 5'- TAT CAT GGA AGC AAG CTC TGT AGG TA -3' | 11506 - 11531 | 1972 |
| VIR13477 | 5'- AGG ATC TTT CAC TCG AGC ACC AAT GT -3' | 13477 - 13452 |  |
| VIF13200 | 5'- AAA TAA GGG GCC TGT CAG CGG AAG AAA AAT -3' | 13200 - 13229 | 1993 |
| VIR15192 | 5'- ACC AAA CAA AGA TTT GGT GAA TGA CAG GA -3' | 15192 - 15164 |  |
| CL+ | 5’- CGC CAG GGT TTT CCC AGT CAC GAC -3’ | / | / |
| CL- | 5’- GTC GTG ACT GGG AAA ACC CTG GCG -3’ | / | / |
| 3SR | 5’- ACC ATC GAT CTC AAG AAC AGC CAG TG -3’ | 414 - 439 | / |
| 5LF | 5’- GGC TAT GCC TGT AGA GGG GAT ATG GAG -3’ | 14222 - 14248 | / |
| 5SF | 5’- CCA TTA AGG ACA TAC CTG AAG CGC A -3’ | 14816 - 14840 | / |

K: G or T
