# Supplementary material for: Phylogenetic, antigenic and biological characterization of pigeon paramyxovirus type 1 circulating in China
Source: Virol J. 2017 Sep 29;14:186. doi: 10.1186/s12985-017-0857-7 (PMC5622419; doi:10.1186/s12985-017-0857-7)
Supplement: Supplementary file 2 — Table S2. Reference NDV strains in this study. (DOCX 52 kb) [file 12985_2017_857_MOESM2_ESM.docx]

**Table S2 Reference NDV strains in this study**

| **Accession number** | **ICPI** | **IVPI** | **Country**  **of origin**^*^ | **Host**^$^ | **Year** | **Senders reference** | **Genotype** | **Fig. 1** | **Fig. 2** | **Fig. 3** | **References^#^** |
| --- | --- | --- | --- | --- | --- | --- | --- | --- | --- | --- | --- |
| AY562991 | / | / |  |  | 1967 | Ulster/67 | I | Y | Y |  | [16] |
| AY935489 | / | / | AU |  | 2001 | AU-1108/01 | I | Y | Y |  | [6] |
| AY935490 | / | / | AU |  | 2002 | AU-1334/02 | I | Y | Y |  | [6] |
| AY935499 | / | / |  |  |  | I-2 | I | Y | Y |  | [17] |
| DQ097394 | / | / |  |  | 1966 | PHY-LMV42/66 | I | Y | Y |  | [2] |
| AF077761 | / | / |  |  | 1946 | LaSota/46 | II | Y | Y |  | [17] |
| DQ060053 | / | / |  |  |  | AQI-ND026 | II | Y | Y |  | [17] |
| EU289028 | / | / |  |  |  | VG/GA | II | Y | Y |  | [17] |
| NC002617 | / | / | US | CK | 1946 | B1/46 | II | Y | Y |  | [17] |
| Y18898 | / | / |  |  |  | clone30 | II | Y | Y |  |  |
| EF201805 | / | / |  |  |  | Mukteswar | III | Y | Y |  | [17] |
| FJ430159 | 1.88 | 2.18 | CN | CK | 2005 | JS/7/05/Ch | III | Y | Y |  | [17] |
| AY741404 | / | / | UK | CK | 1933 | Herts/33 | IV | Y | Y |  | [17] |
| EU293914 | / | / |  |  |  | Italien | IV | Y | Y |  | [17] |
| AY562986 | / | / | US | AN | 1993 | US(FL)/44083/93 | V | Y | Y |  | [16] |
| AY562987 | / | / | US |  | 2002 | US(CA)/211472/02 | V | Y | Y |  | [16] |
| AY562990 | / | / | US | MIX | 1971 | US(FL)/Largo/71 | V | Y | Y |  | [16] |
| AJ880277 | / | / | IT |  | 1982 | IT-227/82 | VI | Y | Y |  | [13] |
| AY175754 | / | / | DE | PI | 1999 | 819/99 1383 | VI |  | Y | Y | [1] |
| AY175770 | / | / | UK | PI | 1999 | 921/99 winc. | VI |  | Y | Y | [1] |
| AY175771 | / | / | UK | PI | 1999 | 814/99 winc | VI |  | Y | Y | [1] |
| AY175773 | / | / | UK | PI | 1999 | 992/99 winc. | VI |  | Y | Y | [1] |
| AY471721 | / | / | AE | FA | 2000 | 60/00 256 1856 | VI |  | Y | Y | [1] |
| AY471722 | / | / | IE | PI | 2000 | 753/00 00-10740 | VI |  | Y | Y | [1] |
| AY471723 | / | / | UK | PI | 2002 | 934/02 B | VI |  | Y | Y | [1] |
| AY471724 | 1.26 | / | IE | PI | 2000 | 623/0 00/9701 | VI |  | Y | Y | [1] |
| AY471726 | / | / | UK | PH | 2000 | 691/00 Liv/Kidn | VI |  | Y | Y | [1] |
| AY471727 | / | / | UK | PI | 2002 | 800/02 | VI |  | Y | Y | [1] |
| AY471728 | / | / | AE | PI | 2000 | 208/00 264144-00 | VI |  | Y | Y | [1] |
| AY471729 | 0.81 | / | BE | PI | 1998 | 1073/98 98/196 VB | VI |  | Y | Y | [1] |
| AY471730 | / | / | UK | PI | 2002 | 799/02 LW 10B4058 | VI |  | Y | Y | [1] |
| AY471731 | / | / | UK | PI | 2002 | 389/02 Preston | VI |  | Y | Y | [1] |
| AY471732 | 0.91 | / | IE | PI | 2001 | 784/01 01-10562 | VI |  | Y | Y | [1] |
| AY471733 | / | / | IE | PI | 2001 | 842/01 01-11068 | VI |  | Y | Y | [1] |
| AY471734 | / | / | UK | PI | 2002 | 389/02 B | VI |  | Y | Y | [1] |
| AY471735 | 1.36 | 0.76 | CA | PI | 1990 | 1606/91 v484/90 | VI |  | Y | Y | [1] |
| AY471736 | / | / | UK | PI | 2001 | 912/01 14B52 | VI |  | Y | Y | [1] |
| AY471738 | / | / | DK | PI | 2000 | 1008/00 72-76767 | VI |  | Y | Y | [1] |
| AY471739 | / | / | DK | PI | 2001 | 34/01 72-80114 | VI |  | Y | Y | [1] |
| AY471740 | / | / | SE | PI | 2000 | 922/00 IF84/00 | VI |  | Y | Y | [1] |
| AY471741 | / | / | IT | CK | 2000 | 463/00 2875/V00 | VI |  | Y | Y | [1] |
| AY471742 | / | / | IT | DO | 2001 | 61/01 252 Vo1 | VI |  | Y | Y | [1] |
| AY471743 | / | / | IE | PI | 2001 | 931/01 01-12630 | VI |  | Y | Y | [1] |
| AY471744 | 1 | / | IE | PI | 2000 | 968/00 PV35/00 | VI |  | Y | Y | [1] |
| AY471745 | / | / | AE | PI | 2000 | 60/00 251-1665-99 | VI |  | Y | Y | [1] |
| AY471746 | / | / | UK | PI | 2000 | 559/00 10-B4032/0600 | VI |  | Y | Y | [1] |
| AY471747 | 0.5 | / | BE | PI | 1998 | 1073/98 98/217VB | VI |  | Y | Y | [1] |
| AY471749 | / | / | UK | PI | 2001 | 21186 | VI |  | Y | Y | [1] |
| AY471751 | / | / | UK | PI | 2002 | 798/02 LW 10B4025 | VI |  | Y | Y | [1] |
| AY471752 | / | / | UK | PI | 2001 | 887/01 2887/b Norwich | VI |  | Y | Y | [1] |
| AY471753 | / | / | UK | PI | 2001 | 924/01 | VI |  | Y | Y | [1] |
| AY471754 | 1.21 | / | UK | PI | 2002 | 974/02 Pv 02 010605 | VI |  | Y | Y | [1] |
| AY471755 | / | / | UK | PI | 2002 | 363/02 D | VI |  | Y | Y | [1] |
| AY471756 | / | / | UK | PI | 2002 | 964/02 3 | VI |  | Y | Y | [1] |
| AY562988 | / | / | US |  | 1972 | US(CA)/1083 Fontana/72 | VI | Y | Y |  | [16] |
| AY562989 | / | / | IT | DO | 2000 | dove/Italy/2736/00 | VI | Y | Y |  | [17] |
| FJ766526 | / | / | CN | PI | 2007 | JS/07/22/Pi | VI |  | Y | Y | [12] |
| FJ766530 | / | / | CN | PI | 2007 | JS/07/04/Pi | VI |  | Y | Y | [12] |
| FJ766531 | / | / | CN | PI | 2007 | JS/07/03/Pi | VI | Y | Y | Y |  |
| GQ281085.1 | / | / | CN | PI | 2007 | JS-35-07-Pi | VI |  | Y | Y |  |
| GQ281086.1 | / | / | CN | PI | 2007 | WX-10-07-Pi | VI |  | Y | Y |  |
| GQ281087.1 | / | / | CN | PI | 2007 | YZ-21-07-Pi | VI |  | Y | Y |  |
| GQ281088.1 | / | / | CN | PI | 2007 | YZ-23-07-Pi | VI |  | Y | Y |  |
| GU551934.1 | / | / | CN | C-PI | 2008 | carrier-pigeon/Guangdong/2008 | VI |  | Y | Y |  |
| HM063423 | / | / | CN | WWH |  | W4 | VI |  | Y | Y | [7] |
| HM063425 | / | / | CN | W-PI |  | P4 | VI | Y | Y | Y | [7] |
| JQ979176 | / | / | CN | PI | 2011 | pi/China/SDLC/2011 | VI | Y | Y | Y | [11] |
| JX244789.1 | / | / | CN | PI | 2000 | Pigeon/China/JM/11/00 | VI |  | Y | Y |  |
| JX244798.1 | / | / | CN | PI | 2008 | Pigeon/China/104/08 | VI |  | Y | Y |  |
| JX244799.1 | / | / | CN | PI | 2008 | Pigeon/China/105/08 | VI |  | Y | Y |  |
| JX244800.1 | / | / | CN | PI | 2008 | Pigeon/China/106/08 | VI |  | Y | Y |  |
| JX486550 | 1.45 | / | CN | PI | 2011 | pi/CH/LGD/110208 | VI | Y | Y | Y | [10] |
| JX486551 | 1.29 | / | CN | PI | 2010 | pi/CH/LJL/100605 | VI |  | Y | Y | [10] |
| JX486552 | 0.63 | / | CN | PI | 2011 | pi/CH/LLN/110713 | VI |  | Y | Y | [10] |
| JX486553 | / | / | CN | PI | 2011 | pi/CH/LHLJ/110813 | VI | Y | Y | Y | [10] |
| JX486554 | 1.19 | / | CN | PI | 2011 | pi/CH/LHLJ/110822 | VI |  | Y | Y | [10] |
| JX486555 | / | / | CN | PI | 2011 | pi/CH/LGD/110945 | VI |  | Y | Y | [10] |
| JX486556 | / | / | CN | PI | 2011 | pi/CH/LGD/110947 | VI |  | Y | Y | [10] |
| JX486557 | / | / | CN | PI | 2012 | pi/CH/LJL/120404 | VI |  | Y | Y | [10] |
| JX901109.1 | / | / | BE |  | 1998 | Belgium/98-238/1998 | VI |  | Y | Y | [20] |
| JX901110.1 | / | / | BE |  | 1998 | Belgium/98-248/1998 | VI |  | Y | Y | [20] |
| JX901111.1 | / | / | BE |  | 1998 | Belgium/98-321/1998 | VI |  | Y | Y | [20] |
| JX901118.1 | / | / | BE |  | 2003 | Belgium/03-05843/2003 | VI |  | Y | Y | [20] |
| JX901120.1 | / | / | BE |  | 2005 | Belgium/05-03936-8/2005 | VI |  | Y | Y | [20] |
| JX901121.1 | / | / | BE |  | 2007 | Belgium/07-04943/2007 | VI |  | Y | Y | [20] |
| JX901122.1 | / | / | BE |  | 2011 | Belgium/11-07574/2011 | VI |  | Y | Y | [20] |
| JX901123.1 | / | / | BE |  | 2011 | Belgium/11-08304/2011 | VI |  | Y | Y | [20] |
| JX901124.1 | / | / | BE |  | 2011 | Belgium/11-09620/2011 | VI |  | Y | Y | [20] |
| KJ808819 | 1.2 | / | CN | PI | 2013 | Pigeon/China/BJ2013 | VI |  | Y | Y | [9] |
| KM374056 | / | / | CN |  | 2013 | pi/YN/1111/13 | VI |  | Y | Y | [8] |
| KM374056 | / | / | CN | PI | 2013 | pigeon/Yunnan/1111/2013 | VI |  | Y | Y | [14] |
| KM374057 | / | / | CN | PI | 2012 | pi/AH/2365/12 | VI | Y | Y | Y | [8] |
| KM374058 | / | / | CN |  | 2012 | pi/AH/2369/12 | VI |  | Y | Y | [8] |
| KM374059 | / | / | CN |  | 2013 | pi/GX/1015/13 | VI |  | Y | Y | [8] |
| KM374060 | / | / | CN |  | 2011 | pi/SH/215/11 | VI |  | Y | Y | [8] |
| KM374061 | / | / | CN |  | 2012 | pi/ZJ/2036/12 | VI |  | Y | Y | [8] |
| KT163261 | / | / | CN | PI | 2012 | Pi/SH/CH/0163/2012 | VI | Y | Y | Y | in this study |
| KT163262 | / | / | CN | PI | 2013 | Pi/SH/CH/0167/2013 | VI | Y | Y | Y | in this study |
| KT163263 | / | / | CN | PI | 2013 | Pi/SH/CH/0168/2013 | VI | Y | Y | Y | in this study |
| KT163264 | / | / | CN | PI | 2012 | Pi/SD/CH/0132/2012 | VI | Y | Y | Y | in this study |
| AF431744 | / | / | CN | GO | 2000 | ZJ-1 | VII | Y | Y |  | [5] |
| DQ485229 | / | / | CN |  | 2002 | CN/Guangxi7/02 | VII | Y | Y |  | [17] |
| DQ485230 | / | / | CN |  | 2003 | CN/Guangxi9/03 | VII | Y | Y |  | [17] |
| DQ485231 | / | / | CN |  | 2003 | CN/Guangxi11/03 | VII | Y | Y |  | [17] |
| DQ659677 | / | / | CN |  |  | NA-1 | VII | Y | Y |  | [19] |
| FJ751918 | / | / |  |  |  | QH1 | VIII | Y | Y |  | [15] |
| FJ436302 | / | / | CN |  |  | F48E8 | IX | Y | Y |  | [17] |
| DQ097393 | / | / |  |  | 1999 | DE-R49/99 | Class I | Y |  |  | [2] |
| FJ794269 | / | / | CN | DU | 2008 | NDV08-004 | Class I | Y |  |  | [4] |
| HQ008337 | / | / | CN |  |  | JS10 | Class I | Y |  |  | [18] |
| JQ713944 | / | / |  |  |  | 9a5b | Class I | Y |  |  | [3] |
| AB070390 | / | / | JP | PI | 1984 | FK-1/84 | VI |  | Y |  | [1] |
| AB070391 | / | / | JP | PI | 1984 | Toyama/84 | VI |  | Y |  | [1] |
| AB070392 | / | / | JP | PI | 1984 | Ibaraki/84 | VI |  | Y |  | [1] |
| AB070393 | / | / | JP | PI | 1984 | Nagano-8/84 | VI |  | Y |  | [1] |
| AB070407 | / | / | JP | PI | 1986 | Tochigi/86 | VI |  | Y |  | [1] |
| AB070409 | / | / | JP | PI | 1988 | Niigata/88 | VI |  | Y |  | [1] |
| AB070412 | / | / | JP | PI | 1991 | Kushiro/91 | VI |  | Y |  | [1] |
| AB070413 | / | / | JP | PI | 1991 | Tokachi/91 | VI |  | Y |  | [1] |
| AB070416 | / | / | JP | PI | 1993 | Ehime/93 | VI |  | Y |  | [1] |
| AB070417 | / | / | JP | PI | 1995 | Kumamoto/95 | VI |  | Y |  | [1] |
| AB070419 | / | / | JP | PI | 1995 | Tochigi/95 | VI |  | Y |  | [1] |
| AB070420 | / | / | JP | PI | 1995 | Utsunomiya/95 | VI |  | Y |  | [1] |
| AB070422 | / | / | JP | PI | 1996 | Shiga/96 | VI |  | Y |  | [1] |
| AB070423 | / | / | JP | PI | 1996 | Fukushima/96 | VI |  | Y |  | [1] |
| AB070426 | / | / | JP | PI | 1997 | Saitama/97 | VI |  | Y |  | [1] |
| AB070434 | / | / | JP | PI | 2000 | Gunma/2000 | VI |  | Y |  | [1] |
| AF001119 | / | / | DE | CK | 1996 | D 85/96 | VIIa |  | Y |  | [1] |
| AF001132 | / | / | CH | CK | 1995 | CH 1/95 | VId |  | Y |  | [1] |
| AF091623 | / | / | FI | GS | 1996 | VIIb/5b | VI |  | Y |  | [1] |
| AF109883 | / | / | CZ | CK | 1996 | VIIc/5c | VI |  | Y |  | [1] |
| AF109885 | / | / | UK | PI | 1984 | VIb/4b | VI |  | Y |  | [1] |
| AF109887 | / | / | GB | PA | 1997 | QGB 506/97 | VIc |  | Y |  | [1] |
| AF218132 | / | / | IT | T-DO | 1994 | IT 147/94 | VIII |  | Y |  | [1] |
| AF234031 | / | / | TW | CK | 1999 | TW 156/99 | VIId |  | Y |  | [1] |
| AF358785 | / | / | CN | PI | 1998 | / | VI |  | Y |  | [1] |
| AY135749 | 1.4 | / | DE | PI | 1994 | 205/95 300/94 | VI |  | Y |  | [1] |
| AY135756 | / | / | CA | Gull | 1990 | V/3c | VI |  | Y |  | [1] |
| AY175722 | / | / | UK | PI | 1999 | / | I |  | Y |  | [1] |
| AY175755 | 1.49 | 0 | DK | PI | 1993 | 654/95 81731 | VI |  | Y |  | [1] |
| AY175759 | / | / | IT | T-DO | 2000 | 434/00 2736 | VI |  | Y |  | [1] |
| AY175766 | / | / | UK | PI | 1984 | 2145/84 | VI |  | Y |  | [1] |
| AY175768 | / | / | UK | PI | 1989 | 1528/89 | VI |  | Y |  | [1] |
| AY175769 | / | / | UK | PI | 1990 | 2162/90 | VI |  | Y |  | [1] |
| AY471725 | / | / | UK | PI | 2002 | 53/02 B | VI |  | Y |  | [1] |
| AY471737 | 0.93 | / | IE | PI | 2002 | 910/02 Pv-02 009344 | VI |  | Y |  | [1] |
| AY471748 | / | / | UK | PI | 1986 | 1402/86 Orig. 2 | VI |  | Y |  | [1] |
| AY471757 | 1.11 | / | DE | PI | 1995 | 205/951 45/94 | VI |  | Y |  | [1] |
| AY471758 | / | / | UK | PI | 1990 | 2675/90 B905/11 | VI |  | Y |  | [1] |
| AY471759 | 1.44 | 0 | AT | PI | 1995 | 878/95 Austria D | VI |  | Y |  | [1] |
| AY471760 | 1.37 | / | AT | PI | 1998 | 1113/98 G-V537/98 | VI |  | Y |  | [1] |
| AY471761 | 0.8 | 0 | DK | PI | 1993 | 654/95 81503 | VI |  | Y |  | [1] |
| AY471762 | 1.34 | 0 | DE | PI | 1994 | 791/94 G 650 | VI |  | Y |  | [1] |
| AY471763 | 0.84 | / | DE | PI | 1994 | 205/95 R4/94 | VI |  | Y |  | [1] |
| AY471764 | / | / | UK | PI | 1994 | 999/94 B527/8 | VI |  | Y |  | [1] |
| AY471765 | / | / | FR | PI | 1998 | 1166/98 98/287 | VI |  | Y |  | [1] |
| AY471766 | / | / | UK | PI | 1998 | 561/98 238/437 | VI |  | Y |  | [1] |
| AY471767 | 0.86 | / | DE | PI | 1994 | 205/95 268/94 | VI |  | Y |  | [1] |
| AY471768 | 1.04 | / | IE | PI | 1996 | 994/96 VF96-3464 | VI |  | Y |  | [1] |
| AY471769 | / | / | IT | PI | 1999 | 546/99 1047/99 | VI |  | Y |  | [1] |
| AY471770 | / | / | DE | CK | 1995 | 1225/95 86/95 | VI |  | Y |  | [1] |
| AY471771 | 1.28 | / | AT | PI | 1997 | 1113/98 1082/97 | VI |  | Y |  | [1] |
| AY471772 | / | / | DE | CO | 1995 | 901/95 92/3488 | VI |  | Y |  | [1] |
| AY471773 | / | / | TR | BU | 1995 | 927/95 Coven 6 | VI |  | Y |  | [1] |
| AY471774 | 1.3 | / | PT | PI | 2001 | 119/01 9085 | VI |  | Y |  | [1] |
| AY471775 | 1.45 | / | PT | PI | 1999 | 1196/99 4859/99 | VI |  | Y |  | [1] |
| AY471776 | / | / | PT | PI | 1998 | 1042/98 6667/98 | VI |  | Y |  | [1] |
| AY471777 | 1.12 | / | PT | PI | 1999 | 1196/99 9111/99 | VI |  | Y |  | [1] |
| AY471778 | / | / | AT | PI | 1997 | 1512/97 GV 867 P1 | VI |  | Y |  | [1] |
| AY471779 | / | / | AE | PI | 1998 | 654/98 157 | VI |  | Y |  | [1] |
| AY471780 | / | / | AE | PI | 1999 | 1017/99 247-1548-99 | VI |  | Y |  | [1] |
| AY471781 | / | / | AE | PI | 1996 | 1381/96 85 | VI |  | Y |  | [1] |
| AY471782 | / | / | AE | PI | 1997 | 767/97 92 251-97 | VI |  | Y |  | [1] |
| AY471783 | / | / | AE | KE | 1998 | 1180/98 180 | VI |  | Y |  | [1] |
| AY471784 | / | / | AE | KE | 1998 | 1180/98 182 | VI |  | Y |  | [1] |
| AY471785 | / | / | AE | KE | 1999 | 12/99 193 | VI |  | Y |  | [1] |
| AY471786 | / | / | SA | PI | 1998 | 316/98 15 | VI |  | Y |  | [1] |
| AY471787 | / | / | IL | SW | 2001 | 655/01 508 | VI |  | Y |  | [1] |
| AY471788 | / | / | IL | PI | 2001 | 655/01 467 | VI |  | Y |  | [1] |
| AY471789 | / | / | AT | PI | 2000 | 1063/00 | VI |  | Y |  | [1] |
| AY471790 | / | / | UK | PI | 1989 | 796/89 ZA | VI |  | Y |  | [1] |
| AY471791 | / | / | UK | PI | 1989 | 936/893 17 L/W | VI |  | Y |  | [1] |
| AY471792 | / | / | UK | PI | 1990 | 1077/90 L/W | VI |  | Y |  | [1] |
| AY471793 | / | / | UK | PI | 1988 | 1799/88 VIC Camb. | VI |  | Y |  | [1] |
| AY471794 | 1.5 | 2.06 | FI | PI | 1992 | 1522/92 F/L | VI |  | Y |  | [1] |
| AY471795 | 1.39 | 0 | CA | PI | 1993 | 746/93 93DC 1349 P1455 | VI |  | Y |  | [1] |
| AY471796 | / | / | CA | PI | 1990 | 1606/91 V685/90 | VI |  | Y |  | [1] |
| AY471797 | / | 2.32 | FR | MIX | 1991 | 1111/92 X469 | VI |  | Y |  | [1] |
| AY471798 | / | / | UK | PI | 1990 | 2209/90 B90-10-90 | VI |  | Y |  | [1] |
| AY471799 | / | / | UK | PI | 1991 | 48/91 M294/90(1)A | VI |  | Y |  | [1] |
| AY471800 | / | / | UK | PI | 1991 | 332/91 B-213/91 | VI |  | Y |  | [1] |
| AY471801 | / | / | UK | PI | 1990 | 2470/90 B1075/10 | VI |  | Y |  | [1] |
| AY471802 | / | / | UK | PI | 1990 | 2369/90 8508/90 | VI |  | Y |  | [1] |
| AY471803 | / | / | UK | PI | 1990 | 835/90 | VI |  | Y |  | [1] |
| AY471804 | / | / | UK | PI | 1990 | 2113/90 B892/90 | VI |  | Y |  | [1] |
| AY471805 | / | / | UK | PI | 1991 | 1816/91 B832/9/91 | VI |  | Y |  | [1] |
| AY471806 | / | / | IE | CK | 1991 | 710/91 PV29/91 | VI |  | Y |  | [1] |
| AY471807 | 1.1 | 0.4 | IE | PI | 1992 | 1243/92 PV 57/92 | VI |  | Y |  | [1] |
| AY471808 | 1.69 | / | IE | CK | 1996 | 898/96 96/3265 | VI |  | Y |  | [1] |
| AY471809 | / | / | IE | CK | 1992 | 1057/92 PV 58/92 | VI |  | Y |  | [1] |
| AY471810 | / | / | UK | PI | 1990 | 763/94 B1205/90 | VI |  | Y |  | [1] |
| AY471811 | / | / | UK | PI | / | 516/94 B504/5 | VI |  | Y |  | [1] |
| AY471812 | / | / | UK | PI | / | 514/94 B108/5 | VI |  | Y |  | [1] |
| AY471813 | / | / | UK | PI | 1993 | 334/93 B3774/93 | VI |  | Y |  | [1] |
| AY471814 | / | / | UK | PI | 1993 | 469/93 B247/5 | VI |  | Y |  | [1] |
| AY471815 | / | / | IE | PI | 1991 | 483/91 | VI |  | Y |  | [1] |
| AY471816 | / | / | UK | PI | 1993 | 216/93 B1082/2 | VI |  | Y |  | [1] |
| AY471817 | 1 | / | UK | DO | 1996 | 503/96 B1099 | VI |  | Y |  | [1] |
| AY471818 | / | / | UK | PH | 1996 | 494/96 pheasant | VI |  | Y |  | [1] |
| AY471819 | / | / | UK | PI | 1990 | 1601/90 718/90 | VI |  | Y |  | [1] |
| AY471820 | / | / | UK | PI | 1988 | 873/88 B540/5/88 | VI |  | Y |  | [1] |
| AY471821 | / | / | UK | PI | 1988 | 1745/88 VIC Winc. | VI |  | Y |  | [1] |
| AY471822 | / | / | UK | PI | 1988 | 1951/88 16/11/88 | VI |  | Y |  | [1] |
| AY471823 | / | / | UK | PI | 1988 | 1186/88 | VI |  | Y |  | [1] |
| AY471824 | / | / | UK | PI | 1986 | 868/86 18/07/86 | VI |  | Y |  | [1] |
| AY471825 | / | / | UK | PI | 1990 | 542/90 VIC Camb. | VI |  | Y |  | [1] |
| AY471826 | / | / | UK | PI | 1989 | 1009/89 | VI |  | Y |  | [1] |
| AY471827 | / | / | UK | PI | 1989 | 1643/89 | VI |  | Y |  | [1] |
| AY471828 | / | / | UK | PI | 1990 | 409/90 B948/2 | VI |  | Y |  | [1] |
| AY471829 | / | / | IE | PI | 1990 | 2510/90 PV80/90 | VI |  | Y |  | [1] |
| AY471830 | / | / | UK | PI | 1988 | 2105/88 VIC Camb. | VI |  | Y |  | [1] |
| AY471831 | / | / | UK | PI | 1988 | 1661/88 VIC S/B | VI |  | Y |  | [1] |
| AY471832 | / | / | UK | PI | 1988 | 2100/88 PMV127 | VI |  | Y |  | [1] |
| AY471833 | / | / | UK | PI | 1986 | 1189/86 B26/9 | VI |  | Y |  | [1] |
| AY471834 | / | / | HU | PI | 1984 | 1052/84 Buda ND55 | VI |  | Y |  | [1] |
| AY471835 | / | / | UK | PI | 1994 | 764/94 B939/6 | VI |  | Y |  | [1] |
| AY471836 | / | / | UK | PI | 1989 | 739/89 B4/9/89 | VI |  | Y |  | [1] |
| AY471837 | 1.21 | 0 | IT | PI | 1994 | 811/94 3095 | VI |  | Y |  | [1] |
| AY471838 | 1.3 | / | IT | PI | 1998 | 655/98 192-98 | VI |  | Y |  | [1] |
| AY471839 | 1.51 | 0.92 | IT | PH | 1995 | 3/96 364/95 | VI |  | Y |  | [1] |
| AY471840 | / | / | IT | PI | 1997 | 387/97 N47/A47/Italy | VI |  | Y |  | [1] |
| AY471841 | 1.02 | / | IT | PI | 1996 | 467/96 Italy 93/96 | VI |  | Y |  | [1] |
| AY471842 | / | / | IT | PI | 1990 | 656/91 108/90 | VI |  | Y |  | [1] |
| AY471843 | 1.32 | 0.45 | IT | T-DO | 1996 | 206/96 12/96 | VI |  | Y |  | [1] |
| AY471844 | 1.26 | 1.81 | AT | PI | 1996 | 254/96 21/96 | VI |  | Y |  | [1] |
| AY471845 | 1.55 | / | AT | PI | 1996 | 1359/96 504/96 | VI |  | Y |  | [1] |
| AY471846 | / | / | IT | DO | 2000 | 783/00 4134/V00 | VI |  | Y |  | [1] |
| AY471847 | / | / | UK | PI | 1984 | 946/84 Redman | VI |  | Y |  | [1] |
| AY471848 | / | / | UK | PI | 1984 | 1368/84 Mavler | VI |  | Y |  | [1] |
| AY471849 | 1.18 | 0 | BE | PI | 1984 | 1156/84 83/1013 | VI |  | Y |  | [1] |
| AY471850 | / | / | IT | PI | 1984 | 38/84 Italian NDV | VI |  | Y |  | [1] |
| AY471851 | / | / | UK | PI | 1983 | 1229/83 RA Jones | VI |  | Y |  | [1] |
| AY471852 | / | / | UK | PI | 1983 | 913/83 VIC Card. | VI |  | Y |  | [1] |
| AY471853 | / | / | UK | R-B | 1984 | 490/84 Rice bran | VI |  | Y |  | [1] |
| AY471854 | / | / | UK | PI | 1986 | 662/86 B185/6/86 | VI |  | Y |  | [1] |
| AY471855 | / | / | UK | PI | 1984 | 418/84 Dafs Edin | VI |  | Y |  | [1] |
| AY471856 | / | / | HK | PI | 1986 | 493/86 191920 | VI |  | Y |  | [1] |
| AY471857 | / | / | IQ | PI | 1978 | 1422/84 BVC/78 | VI |  | Y |  | [1] |
| AY471858 | / | / | UK | CK | 1984 | 697/84 Morrison | VI |  | Y |  | [1] |
| KJ808820 | 1.64 | / | CN | PI | 2012 | Pigeon/China/SD2012 | VI |  | Y |  | [9] |
| KJ920204 | / | / | RU | PI | 2011 | Altai/pigeon/770/2011 | VI |  | Y |  |  |
| AB853926 | / | / | JP | CK | 1969 | chicken/Japan/Osaka/2440/1969 | VI |  | Y |  |  |
| GQ429292 | / | / | IE | PI | 1996 | AV324/96 | VI |  | Y |  |  |
| FJ410147 | / | / | US | PI | 1984 | PPMV-1/Maryland/1984 | VI |  | Y |  |  |
| JN986839 | / | / | IE | PI | 2004 | PPMV-1/pigeon/IE/806/04 | VIb |  | Y |  |  |
| KC013033 | / | / | US | PI | 2006 | NJ/USA/0607/2006 | VI |  | Y |  |  |
| FJ480825.1 | / | / | CN | PI | 2005 | PG/CH/JS/1/05 | VI |  | Y |  |  |
| FJ480826.1 | / | / | CN | PI | 2006 | PG/CH/JS/1/06 | VI |  | Y |  |  |
| JX244790.1 | / | / | CN | PI | 2000 | Pigeon/China/ZQ/17/00 | VII |  | Y |  |  |
| JX244791.1 | / | / | CN | PI | 2006 | Pigeon/China/SD/54/06 | VII |  | Y |  |  |
| JX244792.1 | / | / | CN | PI | 2006 | Pigeon/China/SD/55/06 | VII |  | Y |  |  |
| JX244793.1 | / | / | CN | PI | 2008 | Pigeon/China/93/08 | VI |  | Y |  |  |
| JX244794.1 | / | / | CN | PI | 2008 | Pigeon/China/100/08 | VI |  | Y |  |  |
| JX244795.1 | / | / | CN | PI | 2008 | Pigeon/China/101/08 | II |  | Y |  |  |
| JX244796.1 | / | / | CN | PI | 2008 | Pigeon/China/102/08 | II |  | Y |  |  |
| JX244797.1 | / | / | CN | PI | 2008 | Pigeon/China/103/08 | II |  | Y |  |  |
| JX244801.1 | / | / | CN | PI | 2008 | Pigeon/China/107/08 | VI |  | Y |  |  |
| JX244802.1 | / | / | CN | PI | 2008 | Pigeon/China/109/08 | II |  | Y |  |  |
| JX244803.1 | / | / | CN | PI | 2008 | Pigeon/China/110/08 | VI |  | Y |  |  |
| JX244804.1 | / | / | CN | PI | 2008 | Pigeon/China/111/08 | II |  | Y |  |  |
| JX244805.1 | / | / | CN | PI | 2008 | Pigeon/China/112/08 | VI |  | Y |  |  |
| JX244806.1 | / | / | CN | PI | 2008 | Pigeon/China/113/08 | VII |  | Y |  |  |
| JX244807.1 | / | / | CN | PI | 2008 | Pigeon/China/KP/114/08 | II |  | Y |  |  |
| JX855039.1 | / | / | CN | PI | 2003 | pigeon/China/Shaanxi/g3/2003 | II |  | Y |  | [25] |
| JX855040.1 | / | / | CN | PI | 2006 | pigeon/China/Shaanxi/g10/2006 | VI |  | Y |  | [25] |
| Z12111.1 | / | / | UK | CK | 1966 | Warwick | VIe |  | Y |  | [20] |
| AF402136.1 | / | / | BE |  | 1988 | BG-104/88 | V |  | Y |  | [20] |
| AF402134.1 | / | / | BE |  | 1986 | BG-102/86 | V |  | Y |  | [20] |
| AF001133.1 | / | / |  |  | 1996 | A-24/96 | VI |  | Y |  | [20] |
| AF001131.1 | / | / |  |  | 1995 | S-1/95 | VI |  | Y |  | [20] |
| AF001130.1 | / | / |  |  | 1995 | DK-6/95 | VI |  | Y |  | [20] |
| AF001129.1 | / | / |  |  | 1995 | DK-1/95 | VI |  | Y |  | [20] |
| AF001112.1 | / | / |  |  | 1982 | H-310/82 | V |  | Y |  | [20] |
| AF001111.1 | / | / | IL |  | 1970 | Israel 70 | VI |  | Y |  | [20] |
| AF001110.1 | / | / |  |  | 1970 | Lebanon 70 | VI |  | Y |  | [20] |
| AF001109.1 | / | / | KW |  |  | Kuwait 256 | VI |  | Y |  | [20] |
| AF001108.1 | / | / | IQ |  | 1968 | Iraq AG68 | VI |  | Y |  | [20] |
| AF001107.1 | / | / |  |  | 1972 | H-10/72 | V |  | Y |  | [20] |
| AF001106.1 | / | / | US |  | 1971 | CA 1085/71 | V |  | Y |  | [20] |
| AF001105.1 | / | / | US |  | 1970 | NY 70181/70 | V |  | Y |  | [20] |
| EU477191 | 1.15 | / | US | ECD |  | Eurasian Collared Dove/US/TX3988/2004 | VI |  | Y |  | [21] |
| EU477192 | 1.3 | / | US | ECD |  | Eurasian Collared Dove/US/TX4156/2005 | VI |  | Y |  | [21] |
| EU477193 | / | / | US | ECD |  | Eurasian Collared Dove/US/TX2334/2003 | VI |  | Y |  | [21] |
| EU477195 | 1.13 | / | US | ECD |  | Eurasian Collared Dove/US/TX6295/2006 | VI |  | Y |  | [21] |
| EU477196 | 1.31 | / | US | ECD |  | Eurasian Collared Dove/US/TX6306/2007 | VI |  | Y |  | [21] |
| EU477197 | 1.26 | / | US | ECD |  | Eurasian Collared Dove/US/TX6338/2007 | VI |  | Y |  | [21] |
| EU477199 | / | / | US | ECD |  | Eurasian Collared Dove/US/TX3908/2004 | VI |  | Y |  | [21] |
| EU477201 | / | / | US | ECD |  | Eurasian Collared Dove/US/TX4078/2004 | VI |  | Y |  | [21] |
| EU477200 | 1.09 |  | US | M-DO |  | Mourning Dove/US/TX4048/2004 17.81 | VI |  | Y |  | [21] |
| EU477188 | 0.98 | / | US | PI |  | Dove/US/TX-B2580/2004 | VI |  | Y |  | [21] |
| EU477189 | 1.3 | / | US | PI |  | Pigeon/US/RI166/2000 | VI |  | Y |  | [21] |
| EU477190 | 1.26 | / | US | PI |  | Pigeon/US/TX3503/2004 | VI |  | Y |  | [21] |
| EU477198 | 1.29 | / | US | PI |  | Pigeon/US/TX3377/2004 | VI |  | Y |  | [21] |
| EU477202 | 1.14 | / | US | PI |  | Pigeon/US/TX4142/2005 | VI |  | Y |  | [21] |
| EU477194 | / | / | US | ECD |  | Eurasian Collared Dove/US/TX3817/2004 | VI |  | Y |  | [21] |
| KP780870 | / | / | US | R-PI | 2013 | ND0002270 | VI |  | Y |  |  |
| KP780875 | / | / | US | R-PI | 2013 | ND0003553 | VI |  | Y |  |  |
| KP780876 | / | / | US | R-PI | 2013 | ND0003558 | VI |  | Y |  |  |
| KP780872 | / | / | US | R-PI | 2013 | ND0007186 | VI |  | Y |  |  |
| KP780873 | / | / | US | R-PI | 2013 | ND0007187 | VI |  | Y |  |  |
| KP780871 | / | / | US | R-PI | 2013 | ND0007190 | VI |  | Y |  |  |
| KP780874 | / | / | US | R-PI | 2013 | ND0007199 | VI |  | Y |  |  |
| JX901377.1 | / | / | US | PI | 2009 | PA/USA/0906/2009 | VI |  | Y |  |  |
| JX901376.1 | / | / | US | PI | 2009 | PA/USA/0905/2009 | VI |  | Y |  |  |
| JX901375.1 | / | / | US | PI | 2009 | PA/USA/0904/2009 | VI |  | Y |  |  |
| JX901374.1 | / | / | US | PI | 2009 | OH/USA/0903/2009 | VI |  | Y |  |  |
| JX901373.1 | / | / | US | PI | 2009 | PA/USA/0902/2009 | VI |  | Y |  |  |
| JX901369.1 | / | / | US | PI | 2008 | NJ/USA/0812/2008 | VI |  | Y |  |  |
| JX901368.1 | / | / | US | PI | 2008 | PA/USA/0811/2008 | VI |  | Y |  |  |
| JX901367.1 | / | / | US | PI | 2008 | PA/USA/0810/2008 | VI |  | Y |  |  |
| JX901366.1 | / | / | US | PI | 2008 | PA/USA/0809/2008 | VI |  | Y |  |  |
| JX901365.1 | / | / | US | PI | 2008 | PA/USA/0808/2008 | VI |  | Y |  |  |
| JX901364.1 | / | / | US | PI | 2008 | PA/USA/0807/2008 | VI |  | Y |  |  |
| JX901363.1 | / | / | US | PI | 2008 | PA/USA/0806/2008 | VI |  | Y |  |  |
| JX901362.1 | / | / | US | PI | 2008 | PA/USA/0805/2008 | VI |  | Y |  |  |
| JX901361.1 | / | / | US | PI | 2008 | PA/USA/0804/2008 | VI |  | Y |  |  |
| JX901360.1 | / | / | US | PI | 2008 | PA/USA/0803/2008 | VI |  | Y |  |  |
| JX901359.1 | / | / | US | PI | 2008 | PA/USA/0802/2008 | VI |  | Y |  |  |
| JX901358.1 | / | / | US | PI | 2008 | PA/USA/0801/2008 | VI |  | Y |  |  |
| JX901357.1 | / | / | US | PI | 2007 | PA/USA/0728/2007 | VI |  | Y |  |  |
| JX901356.1 | / | / | US | PI | 2007 | PA/USA/0727/2007 | VI |  | Y |  |  |
| JX901355.1 | / | / | US | PI | 2007 | PA/USA/0726/2007 | VI |  | Y |  |  |
| JX901354.1 | / | / | US | PI | 2007 | PA/USA/0725/2007 | VI |  | Y |  |  |
| JX901353.1 | / | / | US | PI | 2007 | PA/USA/0724/2007 | VI |  | Y |  |  |
| JX901352.1 | / | / | US | PI | 2007 | PA/USA/0723/2007 | VI |  | Y |  |  |
| JX901351.1 | / | / | US | PI | 2007 | NJ/USA/0721/2007 | VI |  | Y |  |  |
| JX901350.1 | / | / | US | PI | 2007 | PA/USA/0720/2007 | VI |  | Y |  |  |
| JX901349.1 | / | / | US | PI | 2007 | MD/USA/0719/2007 | VI |  | Y |  |  |
| JX901348.1 | / | / | US | PI | 2007 | PA/USA/0718/2007 | VI |  | Y |  |  |
| JX901347.1 | / | / | US | PI | 2007 | NY/USA/0717/2007 | VI |  | Y |  |  |
| JX901346.1 | / | / | US | PI | 2007 | PA/USA/0716/2007 | VI |  | Y |  |  |
| JX901345.1 | / | / | US | PI | 2007 | MD/USA/0715/2007 | II |  | Y |  |  |
| JX901344.1 | / | / | US | PI | 2007 | ME/USA/0714/2007 | VI |  | Y |  |  |
| JX901343.1 | / | / | US | PI | 2007 | MN/USA/0713.2/2007 | II |  | Y |  |  |
| JX901342.1 | / | / | US | PI | 2007 | MN/USA/0713/2007 | VI |  | Y |  |  |
| JX901341.1 | / | / | US | PI | 2007 | PA/USA/0712/2007 | VI |  | Y |  |  |
| JX901340.1 | / | / | US | PI | 2007 | PA/USA/0711/2007 | VI |  | Y |  |  |
| JX901339.1 | / | / | US | PI | 2007 | NJ/USA/0710/2007 | VI |  | Y |  |  |
| JX901338.1 | / | / | US | PI | 2007 | OH/USA/0709/2007 | VI |  | Y |  |  |
| JX901337.1 | / | / | US | PI | 2007 | CT/USA/0708/2007 | VI |  | Y |  |  |
| JX901336.1 | / | / | US | PI | 2007 | NC/USA/0707/2007 | VI |  | Y |  |  |
| JX901335.1 | / | / | US | PI | 2007 | PA/USA/0706/2007 | VI |  | Y |  |  |
| JX901334.1 | / | / | US | PI | 2007 | PA/USA/0705/2007 | VI |  | Y |  |  |
| JX901333.1 | / | / | US | PI | 2007 | PA/USA/0704/2007 | VI |  | Y |  |  |
| JX901332.1 | / | / | US | PI | 2007 | MO/USA/0703/2007 | VI |  | Y |  |  |
| JX901331.1 | / | / | US | PI | 2007 | PA/USA/0702.2/2007 | II |  | Y |  |  |
| JX901330.1 | / | / | US | PI | 2007 | PA/USA/0702/2007 | VI |  | Y |  |  |
| JX901329.1 | / | / | US | PI | 2007 | PA/USA/0701/2007 | VI |  | Y |  |  |
| JX901328.1 | / | / | US | PI | 2006 | NJ/USA/0607/2006 | VI |  | Y |  |  |
| JX901327.1 | / | / | US | PI | 2006 | PA/USA/0605/2006 | VI |  | Y |  |  |
| JX901326.1 | / | / | US | PI | 2006 | NJ/USA/0604/2006 | VI |  | Y |  |  |
| JX901325.1 | / | / | US | PI | 2006 | NY/USA/0603/2006 | VI |  | Y |  |  |
| JX901324.1 | / | / | US | PI | 2006 | PA/USA/0602/2006 | VI |  | Y |  |  |
| JX901323.1 | / | / | US | PI | 2006 | PA/USA/0601/2006 | VI |  | Y |  |  |
| JX901320.1 | / | / | US | PI | 2005 | PA/USA/0502/2005 | VI |  | Y |  |  |
| JX901319.1 | / | / | US | PI | 2005 | PA/USA/0501/2005 | VI |  | Y |  |  |
| JX901317.1 | / | / | US | PI | 2002 | USA/0201/2002 | VI |  | Y |  |  |
| JX901315.1 | / | / | US | PI | 2001 | USA/0106/2001 | VI |  | Y |  |  |
| JX901313.1 | / | / | US | PI | 2001 | USA/0102/2001 | VI |  | Y |  |  |
| JX901312.1 | / | / | US | PI | 2001 | USA/0101/2001 | VI |  | Y |  |  |
| AF456439.1 | 52.8 | 1.8 | CN | GO | 1998 | JS/2/98/Go | VIb |  | Y |  | [22] |
| AF458015.1 | 52.8 | 1.64 | CN | MIX | 1997 | ZhJ-3/97 | VIg |  | Y |  | [22] |
| AF458016.1 | 55.2 | 1.66 | CN | MIX | 1986 | ZhJ-2/86 | VIg |  | Y |  | [22] |
| AF458017.1 | 50.4 | 1.79 | CN | MIX | 1998 | Sh-2/98 | VIg |  | Y |  | [22] |
| AF458018.1 | 48 | 1.8 | CN | MIX | 1997 | Sh-1/97 | VIg |  | Y |  | [22] |
| AF458019.1 | 56.4 | 1.81 | CN | MIX | 1997 | XJ-3/97 | VIf |  | Y |  | [22] |
| AF458020.1 | 50.4 | 1.89 | CN | MIX | 1991 | XJ-1/91 | VIf |  | Y |  | [22] |
| AF458021.1 | 49.2 | 1.67 | CN | MIX | 1994 | JX-1/94 | VIf |  | Y |  | [22] |
| AY390314.1 | / | / | CN | PI | 1999 | PK9910 | II |  | Y |  | [23] |
| AY390293.1 | / | / | CN | PI | 1998 | SZb9803 | VI |  | Y |  | [23] |
| AY390292.1 | / | / | CN | PI | 1998 | SZa9803 | VI |  | Y |  | [23] |
| AY390291.1 | / | / | CN | PI | 1996 | PB9601 | VI |  | Y |  | [23] |
| AY390290.1 | / | / | CN | PI | 1999 | NP9904 | VI |  | Y |  | [23] |
| AY390288.1 | / | / | CN | PI | 1997 | YZ9712 | VI |  | Y |  | [23] |

^*^ **Abbreviation of country:**

AE=Arab Emirates; AT=Austria; BE=Belgium; CA=Canada; CH=Switzerland; CN=China; CZ=Czech; DE=Germany; DK=Denmark; FI=Finland; FR=France; HK=Hong kong; HU=Hungary; IE=Ireland; IL=Israel; IQ=Iraq; IT=Italy; JP=Japan; PT=Portugal; Q-GB=Great Britain; SA=Saudi Arabia; SE=Sweden; TR=Turkey; TW=Taiwan; UK=United Kingdom.

^$^ **Abbreviation of host:**

AN=anhinga; BU=Budgerigar; CK=chicken; CO=Cockatoo; C-PI=carrier-pigeon; DO=dove; DU=duck; ECD=Eurasian Collared Dove; FA=Falcon; GO=goose; GS=Goosander; GU=Gull; KE=Kestrel; M-DO=Mourning dove; Mix=mixed species; PA=Parakeets; PH=Pheasant; PI=Pigeon; R-B=Rice bran; R-PI=Rock Pigeon; SW=Swan; T-DO=Turtle dove; TU=Turkey; W-PI=wild pigeon; WWH=white-breasted water hen.

**^#^ References**

[1] Aldous, E. W., C. M. Fuller, J. K. Mynn, and D. J. Alexander. 2004. A molecular epidemiological investigation of isolates of the variant avian paramyxovirus type 1 virus (PPMV-1) responsible for the 1978 to present panzootic in pigeons. Avian Pathol 33:258-269.

[2] Czeglédi, A., D. Ujvari, E. Somogyi, E. Wehmann, O. Werner, and B. Lomniczi. 2006. Third genome size category of avian paramyxovirus serotype 1 (Newcastle disease virus) and evolutionary implications. Virus Res 120:36-48.

[3] Yu, Y., X. Qiu, D. Xu, Y. Zhan, C. Meng, N. Wei, H. Chen, L. Tan, S. Yu, X. Liu, A. Qin, and C. Ding. 2012. Rescue of virulent class I Newcastle disease virus variant 9a5b-D5C1. Virol J 9:120.

[4] Liu, H., F. Chen, Y. Zhao, D. Zheng, J. Li, T. Xu, L. Qi, and Z. Wang. 2010. Genomic characterization of the first class I Newcastle disease virus isolated from the mainland of China. Virus Genes 40:365-371.

[5] Huang, Y., H. Q. Wan, H. Q. Liu, Y. T. Wu, and X. F. Liu. 2004. Genomic sequence of an isolate of Newcastle disease virus isolated from an outbreak in geese: a novel six nucleotide insertion in the non-coding region of the nucleoprotein gene. Brief Report. Arch Virol 149:1445-1457.

[6] Kattenbelt, J. A., M. P. Stevens, and A. R. Gould. 2006. Sequence variation in the Newcastle disease virus genome. Virus Res 116:168-184.

[7] Cai, S., J. Li, M. T. Wong, P. Jiao, H. Fan, D. Liu, M. Liao, J. Jiang, M. Shi, T. T. Lam, T. Ren, and F. C. Leung. 2011. Genetic characterization and evolutionary analysis of 4 Newcastle disease virus isolate full genomes from waterbirds in South China during 2003-2007. Vet Microbiol 152:46-54.

[8] Wang, J., H. Liu, W. Liu, D. Zheng, Y. Zhao, Y. Li, Y. Wang, S. Ge, Y. Lv, Y. Zuo, S. Yu, and Z. Wang. 2015. Genomic Characterizations of Six Pigeon Paramyxovirus Type 1 Viruses Isolated from Live Bird Markets in China during 2011 to 2013. PLoS One 10:e0124261.

[9] Awu, A., M. Y. Shao, M. M. Liu, Y. X. Hu, Z. M. Qin, F. L. Tian, and G. Z. Zhang. 2015. Characterization of two pigeon paramyxovirus type 1 isolates in China. Avian Pathol 44:204-211.

[10] Guo, H., X. Liu, Z. Han, Y. Shao, J. Chen, S. Zhao, X. Kong, and S. Liu. 2013. Phylogenetic analysis and comparison of eight strains of pigeon paramyxovirus type 1 (PPMV-1) isolated in China between 2010 and 2012. Arch Virol 158:1121-1131.

[11] Kumar, C. S., and S. Kumar. 2014. Species based synonymous codon usage in fusion protein gene of Newcastle disease virus. PLoS One 9:e114754.

[12] Song, Q., Y. Cao, Q. Li, M. Gu, L. Zhong, S. Hu, H. Wan, and X. Liu. 2011. Artificial recombination may influence the evolutionary analysis of Newcastle disease virus. J Virol 85:10409-10414.

[13] Ujvari, D., E. Wehmann, J. Herczeg, and B. Lomniczi. 2006. Identification and subgrouping of pigeon type Newcastle disease virus strains by restriction enzyme cleavage site analysis. J Virol Methods 131:115-121.

[14] Wang, J., Y. Lv, Y. Zhang, D. Zheng, Y. Zhao, D. Castellan, H. Liu, and Z. Wang. 2016. Genomic Characterizations of a Newcastle Disease Virus Isolated from Ducks in Live Bird Markets in China. PLoS One 11:e0158771.

[15] Cao, Y., M. Gu, X. Zhang, W. Liu, and X. Liu. 2013. Complete Genome Sequences of Two Newcastle Disease Virus Strains of Genotype VIII. Genome announcements 1.

[16] Wise, M. G., D. L. Suarez, B. S. Seal, J. C. Pedersen, D. A. Senne, D. J. King, D. R. Kapczynski, and E. Spackman. 2004. Development of a real-time reverse-transcription PCR for detection of newcastle disease virus RNA in clinical samples. J Clin Microbiol 42:329-338.

[17] Qiu, X., Q. Sun, S. Wu, L. Dong, S. Hu, C. Meng, Y. Wu, and X. Liu. 2011. Entire genome sequence analysis of genotype IX Newcastle disease viruses reveals their early-genotype phylogenetic position and recent-genotype genome size. Virol J 8:117.

[18] Meng, C., X. Qiu, S. Jin, S. Yu, H. Chen, and C. Ding. 2012. Whole genome sequencing and biological characterization of Duck/JS/10, a new lentogenic class I Newcastle disease virus. Arch Virol 157:869-880.

[19] Liu, J., Y. Cong, R. Yin, C. Ding, S. Yu, X. Liu, C. Wang, and Z. Ding. 2014. The deletion of an extra six nucleotides in the 5 inverted question mark -untranslated region of the nucleoprotein gene of Newcastle disease virus NA-1 decreases virulence. BMC Vet Res 10:305.

[20] Van Borm, S., T. Rosseel, M. Steensels, T. van den Berg, and B. Lambrecht. 2013. What's in a strain? Viral metagenomics identifies genetic variation and contaminating circoviruses in laboratory isolates of pigeon paramyxovirus type 1. Virus Res 171:186-193.

[21] Kim, L. M., D. J. King, H. Guzman, R. B. Tesh, A. P. Travassos da Rosa, R. Bueno, Jr., J. A. Dennett, and C. L. Afonso. 2008. Biological and phylogenetic characterization of pigeon paramyxovirus serotype 1 circulating in wild North American pigeons and doves. J Clin Microbiol 46:3303-3310.

[22] Liu, X. F., H. Q. Wan, X. X. Ni, Y. T. Wu, and W. B. Liu. 2003. Pathotypical and genotypical characterization of strains of Newcastle disease virus isolated from outbreaks in chicken and goose flocks in some regions of China during 1985-2001. Arch Virol 148:1387-1403.

[23] lei, L. H., W. Y. kun, Y. W. wei, Z. G. qiang, and C. F. yan. 2003. [Investigation on the Molecular Epidemiology of Newcastle Disease Virus in China]. Chinese Journal of Veterinary Science:218-221.

[24] Chen, S., Hao, H., Liu, Q., Wang, R., Zhang, P., Wang, X., Du, E., Yang, Z., 2013. Phylogenetic and pathogenic analyses of two virulent Newcastle disease viruses isolated from Crested Ibis (Nipponia nippon) in China. Virus Genes 46, 447-453.

[25] CHEN S, HAO H, LIU Q, et al. Phylogenetic and pathogenic analyses of two virulent Newcastle disease viruses isolated from Crested Ibis (Nipponia nippon) in China [J]. Virus Genes, 2013, 46(3): 447-53.
