# Supplementary material for: Phylogenetic, antigenic and biological characterization of pigeon paramyxovirus type 1 circulating in China
Source: Virol J. 2017 Sep 29;14:186. doi: 10.1186/s12985-017-0857-7 (PMC5622419; doi:10.1186/s12985-017-0857-7)
Supplement: Supplementary file 3 — Table S3. Gene homology of NDV132 to other PPMV-1 strains (DOCX 18 kb) [file 12985_2017_857_MOESM3_ESM.docx]

# Table S3 Gene homology of NDV132 to other PPMV-1 strains

| Subgenotype | clade | strains | Genome | NP | |  | P | |  | M | |  | F | |  | HN | |  | L | |
| --- | --- | --- | --- | --- | --- | --- | --- | --- | --- | --- | --- | --- | --- | --- | --- | --- | --- | --- | --- | --- |
|  |  |  |  | nt | aa |  | nt | aa |  | nt | aa |  | nt | aa |  | nt | aa |  | nt | aa |
| VIb/4bii f | P4-like | **P4** | 97.74 | 98.12 | 98.93 |  | 97.42 | 95.87 |  | 97.56 | 98.61 |  | 97.39 | 98.06 |  | 97.86 | 98.94 |  | 98.27 | 99.18 |
|  |  | JS/07/22/Pi | 98.87 | 99.07 | 99.57 |  | 98.63 | 96.65 |  | 98.42 | 98.89 |  | 99.52 | 99.52 |  | 98.82 | 99.47 |  | 99.02 | 99.27 |
|  |  | pi/CH/LHLJ/110822 | 97.92 | 98.85 | 99.79 |  | 97.32 | 97.18 |  | 98.23 | 99.17 |  | 98.63 | 99.28 |  | 97.19 | 98.41 |  | 98.38 | 99.45 |
|  |  | pi/CH/LGD/110947 | 98.98 | 99.22 | 99.79 |  | 98.98 | 98.47 |  | 99.17 | 99.45 |  | 99.60 | 99.76 |  | 98.82 | 99.12 |  | 99.24 | 99.73 |
| VIb/4bii f | Belgium/98-like | **Belgium/98-238/1998** | 98.21 | 98.71 | 99.15 |  | 97.59 | 96.65 |  | 98.51 | 99.17 |  | 97.97 | 98.31 |  | 98.22 | 98.94 |  | 98.71 | 99.63 |
|  |  | Belgium/98-248/1998 | 98.19 | 98.78 | 99.15 |  | 97.59 | 96.39 |  | 98.51 | 99.17 |  | 97.89 | 98.31 |  | 98.10 | 98.77 |  | 98.69 | 99.54 |
|  |  | Belgium/07-04943/2007 | 96.79 | 97.24 | 98.72 |  | 96.25 | 94.27 |  | 96.30 | 98.61 |  | 96.63 | 97.33 |  | 96.88 | 98.59 |  | 97.64 | 99.13 |
| VIb/4bii f | Belgium/11-like | Pi/SH/CH/0163/2012 | 94.70 | 95.95 | 98.28 |  | 94.13 | 92.92 |  | 94.00 | 98.33 |  | 95.61 | 97.08 |  | 94.10 | 96.80 |  | 95.88 | 98.67 |
|  |  | Pi/SH/CH/0168/2013 | 94.73 | 95.95 | 98.28 |  | 94.13 | 92.92 |  | 94.00 | 98.33 |  | 95.61 | 97.08 |  | 94.16 | 96.80 |  | 95.93 | 98.67 |
|  |  | Belgium/11-07574/2011 | 94.65 | 95.87 | 98.28 |  | 93.65 | 92.65 |  | 94.40 | 98.33 |  | 95.53 | 97.57 |  | 94.29 | 96.62 |  | 95.84 | 98.67 |
|  |  | Belgium/11-09620/2011 | 94.53 | 95.41 | 97.85 |  | 92.77 | 91.00 |  | 94.19 | 97.77 |  | 95.78 | 97.33 |  | 93.70 | 96.43 |  | 95.84 | 98.67 |
|  |  | pi/YN/1111/13 | 94.46 | 95.33 | 98.28 |  | 93.55 | 92.92 |  | 94.10 | 98.33 |  | 95.09 | 96.83 |  | 93.77 | 96.25 |  | 95.85 | 98.85 |
|  |  | pi/AH/2365/12 | 94.38 | 95.79 | 98.28 |  | 93.26 | 91.55 |  | 93.78 | 98.05 |  | 95.18 | 97.08 |  | 93.84 | 96.80 |  | 95.72 | 98.85 |
| VIb/4bii d |  | Pigeon/China/SD2012 | 92.17 | 93.84 | 96.54 |  | 91.15 | 89.89 |  | 92.09 | 95.78 |  | 92.44 | 92.79 |  | 90.79 | 94.23 |  | 94.00 | 97.69 |
| VIb/4bi |  | IT-227/82 | 93.47 | 95.74 | 96.46 |  | 91.94 | 90.44 |  | 93.71 | 96.36 |  | 93.83 | 95.56 |  | 92.65 | 95.16 |  | 95.09 | 97.66 |
| VIe |  | US(CA)/1083 Fontana/72 | 90.95 | 92.22 | 95.61 |  | 89.98 | 87.62 |  | 91.94 | 96.36 |  | 91.13 | 95.75 |  | 89.63 | 93.67 |  | 93.06 | 97.29 |
