# Supplementary material for: Phylogenetic, antigenic and biological characterization of pigeon paramyxovirus type 1 circulating in China
Source: Virol J. 2017 Sep 29;14:186. doi: 10.1186/s12985-017-0857-7 (PMC5622419; doi:10.1186/s12985-017-0857-7)
Supplement: Supplementary file 4 — Table S4. Gene homology of NDV167 to other genotype VI NDV strains. (DOCX 17 kb) [file 12985_2017_857_MOESM4_ESM.docx]

# Table S4 Gene homology of NDV167 to other genotype VI NDV strains

| Subgenotype | clade | strains | Genome | NP | |  | P | |  | M | |  | F | |  | HN | |  | L | |
| --- | --- | --- | --- | --- | --- | --- | --- | --- | --- | --- | --- | --- | --- | --- | --- | --- | --- | --- | --- | --- |
|  |  |  |  | nt | aa |  | nt | aa |  | nt | aa |  | nt | aa |  | nt | aa |  | nt | aa |
| VIb/4bii f | Belgium/11-like | Pi/SH/CH/0163/2012 | 99.94 | 100.00 | 100.00 |  | 100.00 | 100.00 |  | 99.91 | 99.72 |  | 99.92 | 100.00 |  | 100.00 | 100.00 |  | 99.94 | 99.91 |
|  |  | Pi/SH/CH/0168/2013 | 99.93 | 100.00 | 100.00 |  | 100.00 | 100.00 |  | 99.91 | 99.72 |  | 99.92 | 100.00 |  | 99.94 | 100.00 |  | 99.89 | 99.91 |
|  |  | **Belgium/11-07574/2011** | 99.02 | 99.50 | 100.00 |  | 99.07 | 99.24 |  | 98.80 | 99.72 |  | 99.04 | 99.04 |  | 99.36 | 99.12 |  | 99.30 | 99.54 |
|  |  | Belgium/11-09620/2011 | 98.88 | 98.93 | 99.36 |  | 97.93 | 97.44 |  | 98.61 | 99.17 |  | 99.44 | 99.28 |  | 99.06 | 99.65 |  | 99.22 | 99.54 |
|  |  | pi/YN/1111/13 | 99.21 | 99.07 | 99.79 |  | 98.63 | 98.98 |  | 99.45 | 99.72 |  | 98.96 | 98.79 |  | 99.47 | 99.47 |  | 99.48 | 99.73 |
|  |  | pi/AH/2365/12 | 99.24 | 99.72 | 100.00 |  | 98.72 | 97.95 |  | 99.17 | 99.45 |  | 99.36 | 99.52 |  | 99.65 | 100.00 |  | 99.45 | 99.73 |
| VIb/4bii f | Belgium/98-like | **Belgium/98-238/1998** | 96.01 | 97.01 | 98.72 |  | 96.16 | 96.39 |  | 95.22 | 98.89 |  | 96.81 | 97.82 |  | 95.69 | 97.88 |  | 96.86 | 98.76 |
|  |  | Belgium/98-248/1998 | 95.98 | 97.09 | 98.72 |  | 95.98 | 96.13 |  | 95.22 | 98.89 |  | 96.72 | 97.82 |  | 95.57 | 97.70 |  | 96.84 | 98.67 |
|  |  | Belgium/07-04943/2007 | 95.02 | 96.56 | 98.50 |  | 94.78 | 94.54 |  | 93.60 | 98.33 |  | 95.78 | 96.83 |  | 94.67 | 97.52 |  | 96.00 | 98.48 |
| VIb/4bii f | P4-like | Pi/SD/CH/0132/2012 | 94.73 | 95.95 | 98.28 |  | 94.13 | 92.92 |  | 93.90 | 98.05 |  | 95.52 | 97.08 |  | 94.10 | 96.80 |  | 95.88 | 98.67 |
|  |  | P4 | 95.31 | 96.41 | 98.50 |  | 95.43 | 95.07 |  | 94.01 | 98.33 |  | 96.04 | 97.08 |  | 95.19 | 97.16 |  | 96.29 | 98.48 |
|  |  | JS/07/22/Pi | 94.78 | 95.72 | 97.85 |  | 94.40 | 92.65 |  | 93.57 | 97.49 |  | 95.70 | 97.57 |  | 94.49 | 96.62 |  | 95.78 | 98.20 |
|  |  | pi/CH/LHLJ/110822 | 94.21 | 95.95 | 98.07 |  | 93.93 | 93.46 |  | 93.48 | 97.77 |  | 95.27 | 96.83 |  | 93.17 | 95.89 |  | 95.37 | 98.39 |
|  |  | pi/CH/LGD/110947 | 94.62 | 95.72 | 98.07 |  | 94.68 | 94.54 |  | 93.78 | 98.05 |  | 95.61 | 97.33 |  | 93.71 | 95.89 |  | 95.82 | 98.76 |
| VIb/4bii d |  | Pigeon/China/SD2012 | 92.17 | 93.61 | 96.76 |  | 90.96 | 90.44 |  | 91.37 | 96.07 |  | 92.27 | 93.56 |  | 90.76 | 93.49 |  | 94.03 | 97.22 |
| VIb/4bi |  | IT-227/82 | 93.45 | 95.74 | 96.67 |  | 91.64 | 91.00 |  | 93.08 | 96.65 |  | 93.69 | 95.94 |  | 92.50 | 95.34 |  | 94.97 | 97.15 |
| VIe |  | US(CA)/1083 Fontana/72 | 90.84 | 92.47 | 96.04 |  | 89.75 | 89.32 |  | 90.85 | 96.65 |  | 90.98 | 95.56 |  | 89.74 | 93.30 |  | 92.75 | 96.49 |
